# Bactofilin-mediated organization of the ParAB*S* chromosome segregation system in *Myxococcus xanthus*

**DOI:** 10.1038/s41467-017-02015-z

**Published:** 2017-11-28

**Authors:** Lin Lin, Manuel Osorio Valeriano, Andrea Harms, Lotte Søgaard-Andersen, Martin Thanbichler

**Affiliations:** 1 Laboratory for Microbiology, Faculty of Biology, Philipps University, 35043 Marburg, Germany; 2 Max Planck Fellow Group “Bacterial Cell Biology”, Max Planck Institute for Terrestrial Microbiology, 35043 Marburg, Germany; 3 Department of Ecophysiology, Max Planck Institute for Terrestrial Microbiology, 35043 Marburg, Germany; 4 LOEWE Center for Synthetic Microbiology, Hans-Meerwein-Straße, 35043 Marburg, Germany; 5 Present Address: Biozentrum, University of Basel, 4056 Basel, Switzerland

## Abstract

In bacteria, homologs of actin, tubulin, and intermediate filament proteins often act in concert with bacteria-specific scaffolding proteins to ensure the proper arrangement of cellular components. Among the bacteria-specific factors are the bactofilins, a widespread family of polymer-forming proteins whose biology is poorly investigated. Here, we study the three bactofilins BacNOP in the rod-shaped bacterium *Myxococcus xanthus*. We show that BacNOP co-assemble into elongated scaffolds that restrain the ParAB*S* chromosome segregation machinery to the subpolar regions of the cell. The centromere (*parS*)-binding protein ParB associates with the pole-distal ends of these structures, whereas the DNA partitioning ATPase ParA binds along their entire length, using the newly identified protein PadC (MXAN_4634) as an adapter. The integrity of these complexes is critical for proper nucleoid morphology and chromosome segregation. BacNOP thus mediate a previously unknown mechanism of subcellular organization that recruits proteins to defined sites within the cytoplasm, far off the cell poles.

## Abstract (web summary)

The roles played by bactofilins, a widespread type of bacterial cytoskeletal elements, are unclear. Here, the authors show that the bactofilins BacNOP facilitate proper subcellular localization of the ParABS chromosome segregation system in the model organism *Myxococcus xanthus*.

## Introduction

The function of cells critically depends on the proper spatiotemporal organization of their components. In particular, many proteins need to be targeted to distinct subcellular positions to perform localized activities in vital processes such as DNA segregation, cell division, cell polarity, or cell growth. Eukaryotic cells often sort proteins into membrane-bounded organelles to confine their distribution and establish compartments with specialized functions. Bacteria, by contrast, usually lack this compartmentalization mechanism. Nevertheless, they have evolved strategies to organize their cytoplasm into functionally distinct domains, whose maintenance is essential for survival and fitness^1–3^.

In rod-shaped bacteria, specialized subcellular domains are most commonly established at the cell poles. Interestingly, the molecular landmarks guiding the formation of polar domains vary significantly among different bacterial lineages^4–6^. Among the best-studied determinants are the scaffolding proteins DivIVA and PopZ. DivIVA is a coiled-coil protein that is highly conserved among Gram-positive bacteria. It assembles into lattice-like oligomeric structures in vitro^7, 8^ and specifically associates with negatively curved membranes at the cell poles and division septa^9–11^. Depending on the species, these assemblies interact, directly or indirectly, with different proteins to regulate cell division^12, 13^, chromosome segregation^14–18^, and/or cell wall biogenesis^19–21^. PopZ, on the other hand, is limited to Gram-negative alphaproteobacteria. Its homolog from *Caulobacter crescentus* was shown to form branched oligomers in vitro and to self-assemble into a dense matrix that is associated with the cell poles^22–25^. Apart from mediating the polar localization of signaling proteins involved in cell cycle regulation, PopZ also plays a central role in chromosome segregation by controlling the localization and dynamics of the chromosome segregation machinery^22, 23, 26^.

Both PopZ and, in part, DivIVA affect chromosome segregation by interacting with the ParAB*S* DNA partitioning system, a highly conserved module that mediates segregation of the chromosomal replication origin regions in a wide variety of bacteria^27, 28^. ParB is a DNA-binding protein that recognizes conserved sequence (*parS*) motifs clustered within the origin region^29^. In new-born *C. crescentus* cells, a single ParB·*parS* complex is tethered to a large assembly of PopZ that is associated with the old cell pole^22, 23^. At the onset of S-phase, the origin region is released and duplicated. Its two copies immediately re-associate with ParB and then move apart, with one of them reconnecting to PopZ at the old pole and one traversing the cell and attaching to a newly formed PopZ matrix at the opposite (new) cell pole^26, 29–32^. Origin movement is directed by ParA, a Walker-type ATPase that acts as a nucleotide-dependent molecular switch cycling between an ATP-bound, dimeric and an ADP-bound, monomeric state^33–35^. ParA dimers bind non-specifically to the nucleoid and, in addition, interact with the ParB·*parS* complexes, thereby tethering them to the nucleoid surface. ParB, in turn, stimulates the ATPase activity of interacting ParA dimers, inducing their disassembly. As a consequence, the ParB·*parS* complex is loosened from the nucleoid and able to reconnect with adjacent ParA dimers, thereby gradually moving across the nucleoid surface by a ratchet-like mechanism^33–37^. Efficient translocation of the tethered complex was proposed to depend on the elastic properties of the chromosome^38^. Its directionality is determined by a gradient in the concentration of ParA dimers on the nucleoid that is highest in the vicinity of the new pole and gradually decreases towards the moving ParB·*parS* complex^32, 34, 35, 39^. In *C. crescentus*, formation of this gradient depends on the sequestration of free ParA monomers by PopZ and the landmark protein TipN^34, 35^ and, potentially, on localized dimerization of ParA within the polar PopZ matrix^40^.

Several years ago, an additional group of cytoskeletal proteins, called bactofilins, has been identified in bacteria^41, 42^. Bactofilins are widespread among both Gram-positive and Gram-negative bacteria, with many species containing several paralogous copies. They possess a unique β-helical structure^43–46^ and polymerize into polymeric bundles or sheets in the absence of nucleotide cofactors in vitro^41, 42^. Previous studies suggest that these polymers can act in various cellular pathways. In *C. crescentus*, two bactofilin paralogs assemble into a polar scaffold that recruits a peptidoglycan synthase involved in pole morphogenesis^41^. The human pathogen *Helicobacter pylori*, by contrast, employs a single bactofilin to maintain its characteristic helical cell shape^47^, whereas two of these proteins are required to ensure proper flagellar assembly in *B. subtilis* ^48^. Finally, four bactofilin paralogs have been identified in *Myxococcus xanthus*, a model bacterium that has been studied intensively for its ability to translocate on solid surfaces and to aggregate into multi-cellular fruiting bodies under conditions of nutrient deprivation. One of them, BacM, is important for cell shape maintenance^42^. Its paralog BacP, by contrast, has been implicated in the subpolar localization of the Ras-like GTPase SofG, which mediates the proper sorting of two pole-associated ATPases responsible for the extension and retraction of the polar type IV pili^49^.

Apart from its motility machineries, *M. xanthus* has a variety of other intriguing cell biological features, including a very particular organization of its ParAB chromosome partitioning proteins. In this organism, the spatial organization and segregation dynamics of chromosomal DNA are reminiscent of those in *C. crescentus*, with newborn cells containing a single, fully replicated chromosome whose origin and terminus regions are oriented towards the old and new pole, respectively^50^. However, rather than being attached to the poles, the ParB·*parS* complexes localize to distinct sites within the cytoplasm at a distance of about 1 µm from the cell tips. ParA, on the other hand, forms elongated subpolar patches that bridge the gap between the adjacent pole and the origin-associated ParB protein^50, 51^. The molecular mechanism mediating this unique arrangement of the chromosome segregation machinery has so far remained unknown.

In this work, we show that the three bactofilins BacNOP of *M. xanthus* co-assemble into extended scaffolds that stretch the subpolar regions and serve to control the localization of both the ParB·*parS* complex and ParA within the cell. ParB associates with the pole-distal ends of these structures, whereas ParA binds along their entire length, recruited by the newly identified adapter protein PadC. The integrity of this complex is critical for faithful chromosome segregation, indicating a close connection between ParAB localization and function. These findings reveal an additional role for bactofilins in the organization of *M. xanthus* cells. Moreover, they provide evidence for a novel mechanism of subcellular organization in which a cytoskeletal element serves as a molecular ruler to position proteins and DNA at a defined distance from the cell poles.

## Results

### BacNOP form elongated structures at the cell poles

**Fig. 1:**
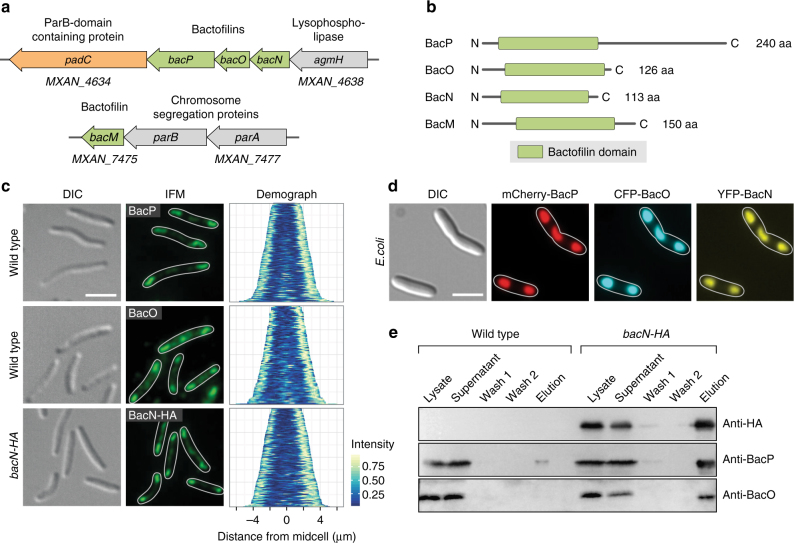
BacNOP co-assemble into extended bipolar structures. **a** Chromosomal context of the four bactofilin genes (*bacM*, *bacN*, *bacO*, and *bacP*) present in the *M. xanthus* DK1622 genome. Arrows indicate the direction of transcription. **b** Domain organization of the *M. xanthus* bactofilin homologs. The bactofilin (DUF583) domain is shown as a green box. Disordered regions are represented by black lines. **c** Subcellular localization of BacP, BacO, and BacN-HA. Cells of strains DK1622 (WT) or LL033 (*bacN::bacN-HA*) were analyzed by immunofluorescence microscopy (IFM), using anti-BacP, anti-BacO (DK1622), or anti-HA (LL033) primary antibodies and an Alexa-Fluor 488-conjugated secondary antibody (bar: 3 µm). In the demographs on the right, the fluorescence profiles of individual cells were sorted according to cell length and stacked on each other, with the shortest cell shown at the top and the longest cell shown at the bottom (*n* = 165 cells for BacP, 100 cells for BacO, and 150 cells for BacN-HA). **d** Heterologous reconstitution of the BacNOP complex in *E. coli*. *E. coli* strain Rosetta(DE3)pLysS bearing plasmids pLL54 (P_T7_-*mCherry-bacP* e*cfp-bacO*) and pPS20 (P_*tet*_-e*yfp-bacN*) was induced with 0.5 mM IPTG (for 3.5 h) and 0.2 µg/ml aTet (for 2 h) to stimulate the synthesis of fluorescently tagged bactofilin variants. Cells were analyzed by differential interference contrast (DIC) and fluorescence microscopy (bar: 3 µm). The Pearson’s correlation coefficients (PCCs) for the patterns observed are 0.95 ± 0.04 (mCherry-BacP/CFP-BacO, *n* = 119 cells) and 0.94 ± 0.06 (mCherry-BacP/YFP-BacN, *n* = 119 cells). Note that despite the use of the strong T7 and *tet* promoters, the bactofilin fusions are only produced at moderate levels (Supplementary Fig. 9). **e** Co-purification of BacN-HA, BacO, and BacP. Cell lysates of strains DK1622 (wild type) and LL033 (BacN-HA) were incubated with anti-HA affinity beads. After isolation of the beads and two washes, interacting proteins were eluted and detected by immunoblot analysis with anti-HA, anti-BacP, and anti-BacO antibodies. Samples of the cell lysates and the supernatants obtained during the isolation and washing steps were analyzed as controls. Full scans of the Western blots are shown in Supplementary Fig. 10

The *M. xanthus* genome contains four bactofilin genes, named *bacN, bacO, bacP*, and *bacM*, respectively^41^. Whereas *bacM* lies immediately downstream of the *parAB* operon, the *bacNOP* genes are located in a separate putative operon with two uncharacterized open reading frames (Fig. 1a). The corresponding products show the typical architecture of bactofilins, comprising a central bactofilin (DUF583) domain that is flanked by short, unstructured N- and C-terminal regions (Fig. 1b). Notably, BacP has a longer C-terminal region than its paralogs, suggesting a distinct functional role for this protein.

Previous studies have shown that BacM has a variable localization pattern, forming either helical cables that extend throughout the cell or rod-like filaments originating at the cell poles^41, 42^. By contrast, BacP consistently assembles into extended subpolar patches at one or both ends of the cell^49^. The clustering of *bacNOP* suggested a functional relationship between the three gene products. To test whether BacNOP co-assembled into a single polymeric structure in vivo, we first reanalyzed the localization pattern of BacP using immunofluorescence microscopy (Fig. 1c, upper panels). We observed that the shortest cells only contained a single full-sized subpolar patch of 1–2 µm length, whereas no or only faint fluorescence was observed on the opposite side of the cell. Longer cells displayed signals in both subpolar regions, which tended to differ slightly in dimension and intensity. Moreover, they frequently displayed an additional BacP patch at their center, the size of which increased with increasing cell length. Because the length of cells closely correlates with their cell cycle stage^50^, these results indicate that cells are born with one mature and one nascent BacP patch, the latter of which gradually grows to full size as the cells elongate. In parallel, a new patch starts to assemble at midcell, which is then split during cytokinesis, explaining the asymmetric distribution of BacP immediately after fission. Analyzing the localization patterns of BacO and a BacN derivative tagged with a hemagglutinin epitope (BacN-HA), we observed very similar localization patterns (Fig. 1c, middle and lower panels). The three proteins thus appear to occupy the same subcellular sites, suggesting that they could indeed co-assemble into a joint structure.

To determine whether BacP, BacO, and BacN in fact bind to each other, we performed colocalization analyses in the heterologous host *Escherichia coli*, a species lacking endogenous bactofilin homologs. When produced together, mCherry-BacP, CFP-BacO, and YFP-BacN formed extended subpolar or midcell patches whose signals were perfectly superimposable (Fig. 1d). These assemblies did not colocalize with the inclusion body-associated chaperone IbpA (Supplementary Fig. 1a) and were permeable to freely diffusible YFP (compare Supplementary Fig. 5a). In addition, they were able to specifically recruit interacting proteins (see below), suggesting that they represent loose networks of BacNOP polymers rather than compact aggregates of misfolded protein. Additional support for a close association between the three bactofilins came from the observation that it was possible to co-purify BacP and BacO with BacN-HA from *M. xanthus* cell lysates using anti-HA affinity beads (Fig. 1e). Moreover, localization studies showed that in *M. xanthus* BacP patches were fragmented and less organized in the absence of BacO. Conversely, BacO localization was severely impaired in a *bacP* mutant, with filaments of varying length projecting from only one of the cell poles (Supplementary Fig. 1b). Loss of BacN or BacM, by contrast, had no effect on the positioning of the remaining bactofilin homologs (Supplementary Fig. 1c). Immunoblot analysis confirmed that BacNOP accumulated independently of each other (Supplementary Fig. 1d). Together, these findings strongly suggest that the three bactofilins interact directly to form a single heteropolymeric scaffold, with BacP constituting its core and BacO contributing to its positioning and integrity. BacN, by contrast, does not seem to have a significant role in the assembly process.

In an attempt to study BacNOP dynamics in live cells, we replaced individual bactofilin genes in *M. xanthus* with hybrids encoding N- or C-terminal fluorescent protein fusions. However, in all cases, the products formed only a single filament per cell that was detached from the cell poles, suggesting that modification of the termini interfered with the proper localization of BacNOP^41^. Notably, however, fusion of BacN to the small HA affinity epitope had no effect on the positioning or biological activity of the structures (as shown below).

### BacNOP mediate the subpolar localization of ParAB*S* in *M. xanthus*

**Fig. 2:**
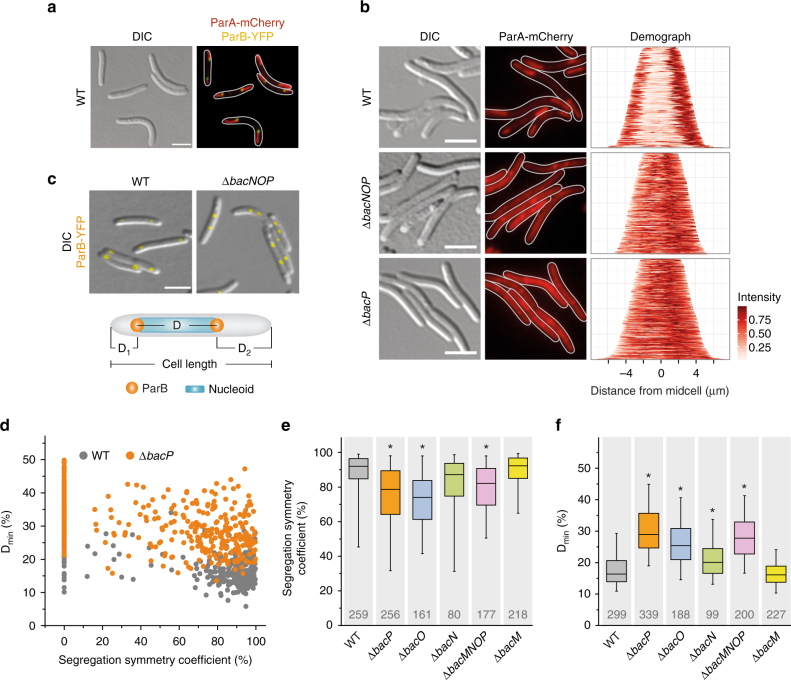
BacNOP are critical for proper localization of the ParAB chromosome segregation proteins. **a** Colocalization of ParA and ParB in *M. xanthus*. Strain LL162 (P_*parA*_-*parA-mCherry P*_*cuoA*_ *-parB-eyfp*) was induced for 20 h with 100 µM CuSO_4_ before imaging. Shown are a DIC micrograph and an overlay of the corresponding mCherry and YFP fluorescence images (bar: 3 µm). **b** Mislocalization of ParA in the absence of BacP. Cells of strains LL145 (P_*parA*_-*parA-mCherry*), LL147 (Δ*bacNOP* P_*parA*_-*parA-mCherry*) and LL152 (Δ*bacP* P_*parA*_-*parA-mCherry*) were analyzed by DIC and fluorescence microscopy (bar: 3 µm). Demographs summarizing the single-cell fluorescence profiles observed for the three strains are given on the right (*n* = 166 cells for WT, 187 cells for Δ*bacNOP*, and 225 cells for Δ*bacP*). **c** Mislocalization of ParB in the absence of BacNOP. Shown are overlays of DIC and fluorescence micrographs of strains LL012 (P_*parB*_-*parB-eyfp*) and LL019 (Δ*bacNOP* P_*parB*_-*parB-eyfp*) (bar: 3 µm). The schematic explains the parameters used for the analysis in panels **d**–**f**. **d** Quantitative analysis of ParB localization in wild-type and Δ*bacP* populations. Cells of strain LL012 (P_*parB*_-*parB-eyfp*) and LL015 (Δ*bacP* P_*parB*_-*parB-eyfp*) were analyzed by DIC and fluorescence microscopy (*n* = 299 cells and 339 cells, respectively). Overlays of the images were used to determine the cell lengths and the distances of the ParB-YFP foci from the cell poles (D_1_ and D_2_; see panel **c**). The segregation symmetry coefficient *S* indicates how symmetrically ParB-YFP foci are arranged within the cell, with *S* = D/(D + D2-D1)x100% and D = cell length-D1-D2. D_min_ gives the smallest distance between a ParB-YFP focus and a cell pole normalized to cell length, with D_min_ = D_1_/cell length × 100%. Note that, by definition, *S* = 0 % for cells containing only a single ParB-YFP focus. **e** and **f** Aberrant segregation and positioning of the ParB·origin complexes in the absence of bactofilins. The segregation symmetry coefficient **e** and D_min_ **f** were determined for strain LL012 (P_*parB*_-*parB-eyfp*) (WT) and its derivatives LL015 (Δ*bacP*), LL018 (Δ*bacO*), LL014 (Δ*bacN*), LL016 (Δ*bacM* Δ*bacNOP*), and LL013 (Δ*bacM*). The data are represented by box plots. The center line shows the median, the box limits indicate the 25th and 75th percentile, and whiskers extend to the 5th and 95th percentile. Only cells containing two ParB-YFP foci were considered in panel **e**. The number of cells analyzed for each strain is given underneath the plots. Significant differences between the wild-type and mutant strains are indicated by asterisks (*p* < 0.0001; Mann–Whitney test)

In *M. xanthus*, ParA and ParB display unique localization patterns, with ParA forming elongated subpolar patches whose distal ends are associated with the origin-bound ParB·*parS* complex (as verified in Fig. 2a). Moreover, additional ParA patches are observed at the cell center during later stages of the cell cycle^50, 51^. The striking similarity between the subcellular distributions of ParA and BacNOP, together with the proximity of the *bacM* and *parAB* genes (Fig. 1a), raised the possibility that bactofilins were functionally associated with the ParAB*S* system. Consistent with this idea, we observed that a ParA-mCherry fusion^50^ failed to form subpolar patches in cells lacking the whole *bacNOP* cluster or only the *bacP* gene (Fig. 2b). In the *bacP* mutant, the typical bipolar pattern of ParA was restored by ectopic expression of a complementing *bacP* copy (Supplementary Fig. 2a), excluding polar effects of the mutation. Deletion of *bacO*, on the other hand, still allowed for the formation of subpolar ParA-mCherry patches (Supplementary Fig. 2b), which however were highly irregular and reminiscent of the BacP structures observed in the Δ*bacO* background (compare Supplementary Fig. 1b). In the absence of BacN, ParA localization was only slightly altered, whereas deletion of *bacM* had no significant effect (Supplementary Fig. 2b). Based on these results, we conclude that bactofilins are necessary for maintaining the proper subcellular arrangement of ParA, with BacP playing a central role in this process.

Next, we analyzed the positioning of ParB in different bactofilin mutants. As previously reported^50, 51^, wild-type cells generally showed one or two ParB-YFP foci that were placed at a distance of about 15–25% of the cell length from the nearest cell pole (Fig. 2c). In cases with two foci, the signals were typically arranged symmetrically within the cell, indicating that origin replication and segregation had finished successfully (Fig. 2c–f). However, in the Δ*bacMNOP* mutant, this highly regular pattern was severely disturbed, as indicated by a significantly lower segregation symmetry coefficient and a considerable increase in the distance (D_min_) of foci from the nearest cell pole. Similar defects were observed when only *bacP* or *bacO* was deleted. Other bactofilin single-mutants, by contrast, showed only minor (Δ*bacN*) or no (Δ*bacM*) changes in ParB-YFP localization (Fig. 2d–f). Thus, formation of subpolar BacNOP assemblies is critical for proper positioning of the ParB·*parS* complexes.

The involvement of BacNOP in ParAB positioning pointed to an interaction between these proteins. Colocalization analysis revealed that ParB-YFP was indeed consistently detected at the pole-distal ends of the BacNOP structures (Supplementary Fig. 2c and d). Moreover, when cells were treated with the division inhibitor cephalexin, they formed extensive non-polar BacNOP assemblies with ParB-YFP foci positioned at both of their ends (Supplementary Fig. 2e). These findings suggested that ParB specifically associates with the terminal regions of the bactofilin structures. To further test this possibility, we performed pull-down experiments on crude cell extracts of *M. xanthus* wild-type cells using purified StrepII-ParB as a bait. We found that BacP was retained on affinity beads loaded with ParB but not on control beads lacking immobilized protein (Supplementary Fig. 2f), supporting a role of BacP in ParB recruitment. However, subsequent in vitro analyses did not provide any evidence for a direct association between the two proteins (Supplementary Fig. 2g). Similarly, bactofilin polymers did not appear to bind directly to ParA (see below). These results implied the existence of additional, as-yet unknown factors that mediate the recruitment of ParAB to the BacNOP patches.

### BacNOP structures interact with the novel ParB-like protein PadC

**Fig. 3:**
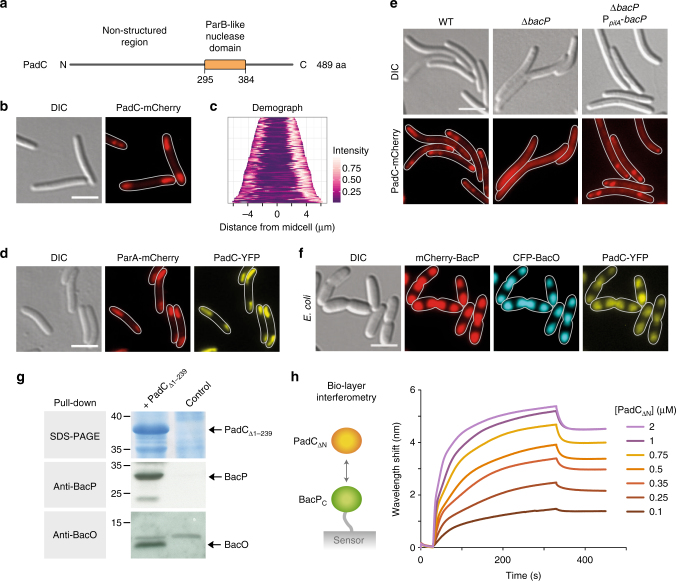
BacP interacts with the ParB-like nuclease domain-containing protein PadC. **a** Domain organization of PadC. The ParB-like nuclease (ParB_C_) domain is indicated in orange. Numbers indicate its position within the polypeptide chain. **b** Subcellular localization of PadC in *M. xanthus*. Cells of strain LL134 (P_*cuoA*_ *-padC-mCherry*) were induced overnight with 200 µM CuSO_4_ and analyzed by DIC and fluorescence microscopy (bar: 3 µm). **c** Demograph showing the subcellular distribution of PadC-mCherry in strain LL134 (see panel **b**; *n* = 109 cells). **d** Colocalization of PadC with ParA in *M. xanthus*. Strain LL201 (P_*parA*_-*parA-mCherry* P_*van*_ *-padC*-*eyfp*) was induced for 2 h with 5 µM vanillate before imaging (bar: 3 µm). **e** Dependence of PadC localization on the presence of BacP. Cells of strains LL116 (*padC*-*mCherry*), LL130 (Δ*bacP padC-mCherry*), and LL135 (Δ*bacP* P_*pilA*_ *-bacP padC-mCherry*) were analyzed by DIC and fluorescence microscopy (bar: 3 µm). **f** Heterologous reconstitution of the BacP·BacO·PadC complex in *E. coli*. Cells of *E. coli* BL21(DE3) bearing plasmids pLL54 (P_T7_-*mCherry-bacP cfp-bacO*) and pLL101 (P_T7_-*padC-eyfp*) were induced for 1.5 h with 0.5 mM IPTG before imaging. (bar: 3 µm). The PCC for the mCherry-BacP and PadC-YFP signals is 0.92 ± 0.03 (*n* = 50 cells). **g** Co-purification of BacP and BacO with PadC. A whole-cell lysate of wild-type strain DK1622 was incubated with Ni-NTA beads loaded with purified His_6_-PadC_Δ1-239_ (+PadC). After isolation of the beads, bound protein was eluted and subjected to SDS–PAGE and to immunoblot analysis with anti-BacP and anti-BacO antibodies, respectively. A reaction with beads not pre-incubated with purified protein served as a control. A molecular mass standard (in kDa) is given on the left. Arrows indicate the positions of the target proteins. Full scans of the SDS-gel and the Western blots are shown in Supplementary Fig. 14. **h** Bio-layer interferometric analysis of the interaction between PadC and BacP. Sensors loaded with biotinylated BacP_Δ1−115_ (BacP_C_) were probed with the indicated concentrations of PadC_Δ1-281_ (PadC_ΔN_). The interaction kinetics were followed by monitoring the wavelength shifts resulting from changes in the optical thickness of the sensor surface during association or dissociation of the analyte. The extent of non-specific binding of PadC_ΔN_ to the sensor surface was negligible (Supplementary Fig. 5d)

**Fig. 4:**
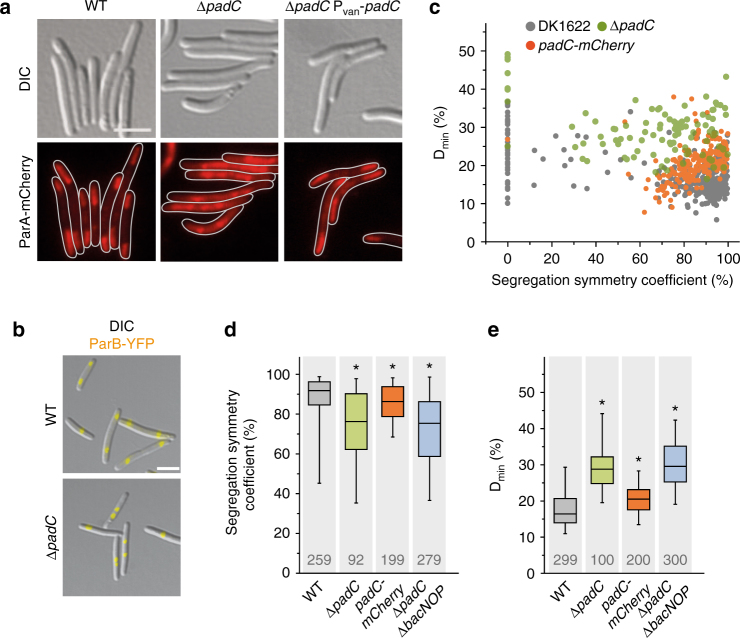
PadC is required for the subpolar localization of ParA and ParB. **a** Mislocalization of ParA in the absence of PadC. Cells of strains of LL145 (P_*parA*_-*parA-mCherry*), LL154 (Δ*padC* P_*parA*_-*parA-mCherry*) and LL192 (Δ*padC* P_*parA*_−*parA−mCherry* P_*van*_ *−padC*) were analyzed by DIC and fluorescence microscopy. LL192 was induced for 2 h with 5 µM vanillate before imaging (bar: 3 µm). **b** Mislocalization of ParB in the absence of PadC. Cells of strains LL012 (P_*parB*_ *-parB-eyfp*) and LL102 (Δ*padC P*_*parB*_ *-parB-eyfp*) were visualized by DIC and fluorescence microscopy. Shown is an overlay of the two images (bar: 3 µm). **c** Quantitative analysis of ParB localization in strains expressing wild-type or mutant *padC* alleles. Cells of strains LL012 (P_*parB*_-*parB-eyfp*), LL102 (*ΔpadC* P_*parB*_-*parB-eyfp*) and LL118 (*padC-mCherry* P_*parB*_-*parB-eyfp*) were analyzed by DIC and fluorescence microscopy (*n* = 299 cells for LL012, 100 cells for LL102, and 200 cells for LL118). Overlays of the images were used to determine D_min_ and the segregation symmetry of ParB-YFP foci (as described for Fig. 2d). **d** and **e** Aberrant segregation and positioning of the ParB·origin complexes in *padC* and bactofilin mutants. The segregation symmetry (K) and D_min_ (L) were determined for strain LL012 (P_*parB*_-*parB-eyfp*) (WT) and its derivatives LL102 (Δ*padC* P_*parB*_-*parB-eyfp*), LL118 (*padC-mCherry* P_*parB*_-*parB-eyfp*), and LL176 (Δ*padC* Δ*bacNOP* P_*parB*_-*parB-eyfp*). Values are represented by box plots (defined in the legend to Fig. 2e and f). Only cells containing two ParB-YFP foci were considered in panel d. The number of cells analyzed for each strain is given. Significant differences between the wild-type and mutant strains are indicated by asterisks (*p* < 0.0001; Mann–Whitney test)

In search of a potential adapter protein, we turned our attention to MXAN_4634, an uncharacterized open reading frame located immediately downstream of *bacNOP* (Fig. 1a). Its predicted gene product features a long disordered N-terminal region and a C-terminal segment that includes a ParB-like nuclease (ParB_C_) domain (Fig. 3a). ParB_C_ domains are typically found in chromosome partitioning proteins of the ParB family, where they mediate the interaction with the centromeric *parS* sites, lacking nuclease activity^33^. The clustering of *bacNOP* and MXAN_4634, hereafter referred to as *padC* (*Pa*rB_C_ *d*omain-*c*ontaining protein), is conserved among various members of the *Myxococcales*, suggesting a functional connection between these genes (Supplementary Fig. 3). To test for a role of PadC in bactofilin function, we first determined the subcellular localization of the protein. Intriguingly, in a merodiploid strain, PadC-mCherry showed the same bipolar distribution as BacNOP and ParA (Figs. 3b and c). Colocalization studies verified that the signals produced by PadC-YFP and ParA-mCherry are indeed perfectly superimposable, suggesting that PadC could be part of the bactofilin·ParA complex (Fig. 3d). To test this possibility, we determined the subcellular distribution of PadC in various bactofilin mutant backgrounds, using strains that carried a *padC-mCherry* fusion in place of the wild-type *padC* gene (Fig. 3e and Supplementary Fig. 4a and b). Of note, in the absence of the wild-type protein, the fusion often formed polar or sub-polar foci (instead of coherent patches) that were localized to the ends of the bactofilin structures, in line with the finding that the tagged protein is only partially functional (see Fig. 4c–e and below). Importantly, however, upon deletion of the whole *bacNOP* cluster (Supplementary Fig. 4a) or only the *bacP* gene (Fig. 3e), PadC-mCherry lost this localization pattern and became evenly distributed within the cell. This effect was fully reversed by expression of a complementing copy of *bacP* in the Δ*bacP* mutant, excluding any polar effects of the mutation. In the absence of *bacO*, PadC-mCherry still formed foci, which were however mislocalized, whereas no major changes were observed in Δ*bacN* cells (Supplementary Fig. 4b). Western blot analysis showed that mutations in the *bacNOP* genes did not affect the level of PadC (Supplementary Fig. 4c). These results suggest that PadC is recruited to the bactofilin patches through interaction with BacP.

To determine whether PadC can directly bind to bactofilin complexes, we analyzed the ability of PadC-YFP to associate with a complex of mCherry-BacP and CFP-BacO in *E. coli*. A PadC-YFP fusion indeed perfectly colocalized with the bactofilin structures (Fig. 3f), whereas YFP alone did not show any apparent affinity for them (Supplementary Fig. 5a). A similar result was obtained for a truncated variant of PadC lacking the disordered N-terminal region (Venus-PadC_Δ1-239_), suggesting that PadC may be recruited to the bactofilin patches through its C-terminal ParB_C_ domain (Supplementary Fig. 5b). This notion is supported by the finding that BacO and BacP can be pulled down from whole-cell extracts of *M. xanthus* wild-type cells using affinity beads loaded with hexahistidine-tagged PadC_Δ1-239_ (Fig. 3g). To verify the interaction between bactofilins and PadC, we aimed to perform in vitro binding studies with purified components. The above results indicated that BacP was necessary and sufficient to recruit PadC. However, due to its tendency to form large polymeric assemblies, the full-length protein was not amenable to quantitative biochemical analyses. A distinctive feature of BacP is its unusually long C-terminal extension (Fig. 1b). As the bactofilin domains of BacNOP are highly similar, we hypothesized that the determinants specifically recognized by PadC may be located in this unique, disordered region. To test this idea, we purified a C-terminal fragment of BacP (BacP_C_) and analyzed it for its binding to an N-terminally truncated variant of PadC (PadC_ΔN_) using bio-layer interferometry (Fig. 3h and Supplementary Fig. 5c and d). Titration experiments revealed that the two fragments indeed interacted with high affinity (*K*_*D*_ *= *340 nM). Collectively, these results demonstrate that PadC associates with bactofilin complexes both in vivo and in vitro.

### PadC is required for proper ParAB*S* positioning

Having identified PadC as a new interactor of the BacNOP complex, we explored whether this protein could serve as an adapter recruiting ParA to the bactofilin patches. In support of this notion, ParA-mCherry lost its typical bipolar distribution in a Δ*padC* mutant and instead accumulated over the nucleoids, often forming distinct foci that could reflect its interaction with ParB·*parS* complexes (Fig. 4a). This phenotype was reversed by expressing a complementing copy of *padC*, excluding any polar effects of the mutation (Fig. 4a). Prompted by this finding, we further tested for a role of PadC in ParB localization. Quantitative analysis of the positions of ParB-YFP foci in Δ*padC* cells revealed a severe defect in the positioning of the chromosomal origin region (Fig. 4b–e), similar to that observed in the Δ*bacP* background (compare Fig. 2e and f). Importantly, concomitant deletion of *padC* and *bacNOP* did not produce a synthetic phenotype (Fig. 4d and e). These results indicate that PadC cooperates with bactofilin complexes to properly localize the ParAB*S* chromosome partitioning machinery. Of note, we observed that deletion of *padC* appeared to affect the integrity of the bactofilin patches (Supplementary Fig. 5e). PadC may thus mediate the positioning of ParB both by controlling the subcellular arrangement of ParA and by ensuring the correct assembly of bactofilin patches at the two cell poles.

### PadC interacts with ParA

**Fig. 5:**
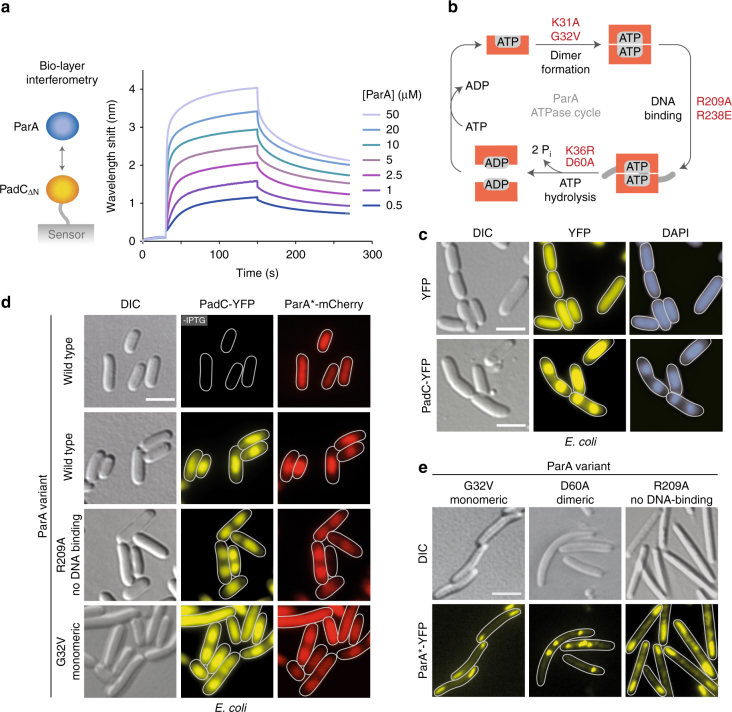
PadC interacts with ParA. **a** Bio-layer interferometric analysis of the interaction between PadC and ParA. Sensors loaded with biotinylated PadC_Δ1-281_ (PadC_ΔN_) were probed with the indicated concentrations of ParA. The extent of non-specific binding of ParA to the sensor surface was negligible (Supplementary Fig. 5d). **b** Putative ATPase cycle of ParA. Monomeric ParA binds ATP and dimerizes. The dimeric complex is able to interact non-specifically with chromosomal DNA. Spontaneous or ParB-stimulated ATP hydrolysis leads to dissociation of the ParA dimer and nucleotide exchange, thereby restarting the cycle. Mutations affecting specific steps of the ParA ATPase cycle are indicated in red. **c** DNA-binding activity of PadC. Cells of *E. coli* BL21(DE3) were transformed with plasmids pLL137(P_T7_-*eyfp*) or pLL101 (P_T7_-*padC-eyfp*) and induced for 4 h with 0.5 mM IPTG before imaging. DNA was stained with DAPI (bar: 3 µm). The PCC for the DAPI and PadC-YFP signals is 0.94 ± 0.04 (*n* = 47 cells). **d** Colocalization of PadC with different ParA variants in *E. coli*. Cells of *E. coli* BL21(DE3) bearing pLL101 (P_T7_-*padC-eyfp*) were transformed with pLL100 (P_*tet*_-*parA-mCherry*), pLL124 (P_*tet*_-*parA*_*R209A*_ *-mCherry*), or pLL172 (P_*tet*_-*parA*_*G32V*_ *-mCherry*) and induced with 0.5 mM IPTG (for 2 h) and/or 0.2 µg/ml aTet (for 1 h) before imaging (bar: 3 µm). The PCCs for the PadC-YFP and ParA^*^-mCherry signals are 0.93 ± 0.05 (WT, *n* = 52 cells), 0.92 ± 0.11 (R209A, *n* = 52 cells), 0.90 ± 0.10 (G32V, *n* = 51 cells). **e** Subcellular localization of mutant ParA variants in *M. xanthus*. Cells of strain LL211 (P_*van*_ *-parA*_*G32V*_ *-eyfp*), LL218 (P_*van*_ *-parA*_*D60A*_ *-eyfp*), or LL193 (P_*van*_ *-parA*_*R209A*_ *-eyfp*) were induced for 5.5 h with 3 µM vanillate before imaging (bar: 3 µm)

Because PadC was colocalized with ParA and required for the recruitment of ParA to the BacNOP complexes, we aimed to test for a direct interaction between the two proteins in vitro (Fig. 5a). As observed for other ParA orthologs^38^, ParA from *M. xanthus* was only soluble in the presence of ATP, which restricted biochemical analyses to the dimeric form of the protein (see also Fig. 5b). Bio-layer interferometric analysis showed that PadC_ΔN_ and purified ParA·ATP indeed tightly bind to each other (*K*_*D*_ = 0.9 µM), supporting a direct role of PadC in regulation of ParA localization. To further investigate the interplay between these two proteins, we turned to in vivo interaction studies. Interestingly, when produced heterologously in *E. coli*, PadC-YFP associated with the chromosomal DNA, leading to strong nucleoid condensation, whereas no such effect was observed upon synthesis of YFP alone (Fig. 5c). This observation is consistent with the presence of a potential, although low-scoring, helix-turn-helix motif in the conserved ParB_C_ domain of PadC (amino acids 346–367). Upon co-production of PadC-YFP and ParA-mCherry, the two fusions colocalized on the condensed nucleoids (Fig. 5d). However, due to the non-specific DNA-binding activity of ParA (Supplementary Fig. 6a; wild type), it was difficult to draw conclusions on the ability of the proteins to interact with each other. To solve this issue, we generated ParA-mCherry variants with substitutions (R209A and R238E) in conserved residues shown to be involved in DNA binding^52^ (Fig. 5b). These variants no longer associated with the chromosome in *E. coli* (Supplementary Fig. 6a). However, upon co-expression with PadC-YFP, they again localized to the condensed nucleoids (Fig. 5d and Supplementary Fig. 6b), demonstrating a direct interaction between ParA-mCherry and the DNA-bound PadC-YFP fusion.

ParA cycles between a monomeric and dimeric state, dependent on nucleotide binding and hydrolysis (Fig. 5b). To clarify how the interaction pattern of ParA correlates with its oligomerization state, we made substitutions in the protein that were predicted to block its ATPase cycle at the steps of dimerization (K31A and G32V) or nucleotide hydrolysis (K36R and D60A), based on previous studies of other ParA homologs^31, 33, 53, 54^. As expected, the K31A and G32V variants lacked non-specific DNA-binding activity when synthesized in *E. coli* (Supplementary Fig. 6a). Nevertheless, they localized to the condensed nucleoids upon co-expression with PadC-YFP, demonstrating that PadC is able to interact with ParA monomers (Fig. 5d and Supplementary Fig. 6b). This result was corroborated by ectopic expression of alleles encoding mutant ParA-YFP fusions in *M. xanthus* cells, which showed that both monomeric variants adopted the typical bipolar pattern observed for PadC (Fig. 5e and Supplementary Fig. 6c; compare Fig. 3d). The ATP-locked, dimeric K36R and D60A variants, on the other hand, exhibited strong DNA-binding activity in *E. coli* (Supplementary Fig. 6a). When expressed in *M. xanthus*, they lacked the typical bipolar distribution and instead formed a variable number of distinct foci (Fig. 5e and Supplementary Fig. 6c), likely positioned over the nucleoid. These results indicate that the bactofilin·PadC complex mostly associates with the monomeric form of ParA in vivo. Interestingly, however, DNA binding-defective variants of ParA (R209A and R238E) also colocalized with PadC (Figs. 5d and e and Supplementary Fig. 6b), even though a sizable fraction of these proteins may be in the dimeric state. Similarly, a constitutively dimeric ParA-mCherry variant defective in DNA binding (D60A R238E) (Supplementary Fig. 6a) still colocalized with PadC-YFP in *E. coli* (Supplementary Fig. 6b). Consistent with the in vitro data (see Fig. 5a), PadC is thus also capable of interacting with ParA dimers, although this ParA species may be largely sequestered to the nucleoid and/or the origin-bound ParB complexes under normal conditions.

### PadC recruits ParA to bactofilin structures

**Fig. 6:**
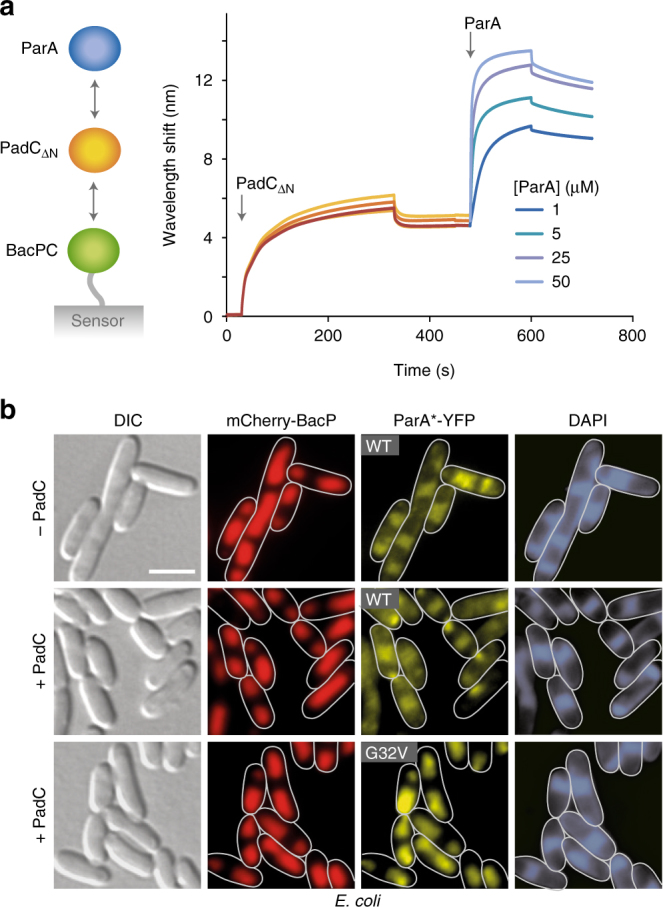
PadC recruits ParA to bactofilin polymers. **a** In vitro reconstitution of the ternary BacP·PadC·ParA complex. Bio-layer interferometry sensors loaded with biotinylated BacP_Δ1-115_ (BacP_C_) were first incubated with 5 µM PadC_Δ1-281_ (PadC_ΔN_). After the dissociation of loosely bound protein, the sensors were transferred into solutions containing the indicated concentrations of ParA (arrow) to monitor the interaction of ParA with the BacP_C_·PadC_ΔN_ complex. The extent of non-specific binding of ParA to BacP_C_-loaded sensors was negligible (Supplementary Fig. 7a). **b** PadC-mediated recruitment of ParA to bactofilin complexes in the heterologous host *E. coli*. Cells of *E. coli* BL21(DE3) bearing plasmid pLL54 (P_T7_-*mCherry-bacP cfp-bacO*) were transformed with plasmid pLL86 (P_*tet*_-*parA-eyfp*) (WT) or pLL215 (P_*tet*_-*parA*_*G32V*_ *-eyfp*) (G32V) and, when indicated (+PadC), with plasmid pLL205 (P_T7_-*padC*). Transformants were induced with 0.5 mM IPTG (for 3.5 h) and 0.2 µg/ml aTet (for 2.5 h) before imaging (bars: 3 µm). The PCCs for the mCherry-BacP and ParA*-YFP signals are 0.1 ± 0.27 (WT without PadC, *n* = 115 cells), 0.51 ± 0.23 (WT with PadC, *n* = 114 cells), and 0.93 ± 0.06 (G32V with PadC, *n* = 97 cells)

The above results show that ParA binds to the ParB_C_ domain of PadC. To further clarify the role of this interaction, we explored whether PadC was sufficient to mediate the recruitment of ParA to bactofilin structures. As a first approach, we set out to reconstitute a ternary BacP·PadC·ParA complex in vitro. To this end, a fragment comprising the C-terminal extension of BacP (BacP_C_) was immobilized on a bio-layer interferometry sensor and incubated with PadC_ΔN_. Subsequent titration of the sensors with purified ParA led to the concentration-dependent formation of a stable ternary complex (Fig. 6a). By contrast, no interaction was observed in control reactions lacking PadC_ΔN_ (Supplementary Fig. 7a), supporting the idea that PadC functions as an adapter mediating the bactofilin–ParA interaction. To validate this hypothesis, we tested for the ability of PadC to recruit ParA-YFP to a complex of mCherry-BacP and CFP-BacO after heterologous expression in *E. coli* (Fig. 6b). Consistent with the above results (Supplementary Fig. 6a), wild-type ParA-YFP was quantitatively associated with the nucleoids in cells lacking PadC (Fig. 6b; top row). By contrast, the fusion became partly associated with the bactofilin structures upon co-expression of the *padC* gene (Fig. 6b, middle row). When the same analysis was repeated with a monomeric, DNA binding-deficient variant (G32V) of ParA-YFP, the protein completely colocalized with the bactofilin structures (Fig. 6b, bottom row), whereas a control strain producing YFP instead of the fusion protein displayed even fluorescence throughout the cell (Supplementary Fig. 7b). In the absence of PadC, the monomeric variant was largely dispersed within the cell, although a minor fraction appeared associated with the bactofilin complexes (Supplementary Fig. 7c). Together, these results strongly support the notion that PadC is necessary and sufficient to recruit ParA, and in particular its monomeric form, to the BacNOP complexes.

### Defects in BacNOP or PadC affect chromosome structure and segregation

**Fig. 7:**
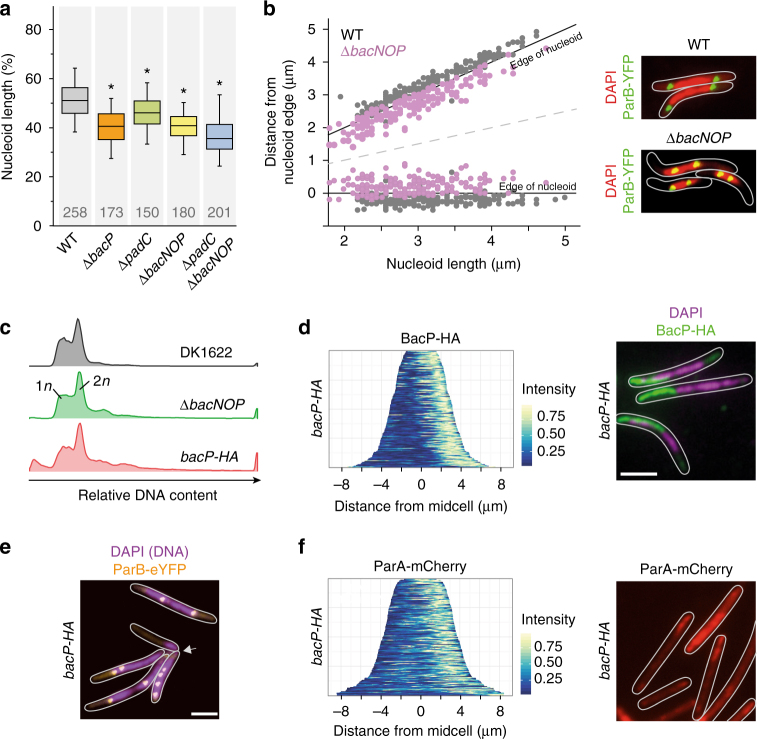
The BacNOP·PadC complex functions in nucleoid organization and DNA segregation. **a** Shortening of the nucleoids in the absence of bactofilins or PadC. Strains DK1622 (WT), LL001 (Δ*bacP*), LL101 (Δ*padC*), MT295 (Δ*bacNOP*), and LL174 (Δ*bacNOP* Δ*padC*) were stained with DAPI and analyzed by DIC and fluorescence microscopy. Shown is the length of the nucleoid along the longitudinal axis of the cell normalized to cell length. Values are represented as box plots (defined in the legend to Fig. 2e and f). The number of cells analyzed for each strain is given. Significant differences between the wild-type and mutant strains are indicated by asterisks (*p* < 0.001; *t-*test). **b** Aberrant positioning of the chromosomal origin regions in the absence of BacNOP. Cells of strains LL012 (P_*parB*_-*parB-eyfp*) and LL019 (Δ*bacNOP* P_*parB*_-*parB-eyfp*) were analyzed by fluorescence microscopy to determine the positions of ParB-YFP foci relative to the edges of the nucleoid, as visualized by DAPI staining (*n* = 179 cells for LL012 and 181 cells for LL019). Images of exemplary cells are given on the right. **c** Changes in DNA content upon mutation of bactofilin genes. Cells of strains DK1622 (WT), MT295 (Δ*bacNOP*), and LL032 (*bacP-HA*) were incubated with a fluorescent DNA stain and subjected to flow cytometric analysis. Shown are histograms giving the distribution of fluorescence intensities in the different cell populations (*n* = 30,000 cells per strain). **d** Asymmetric subcellular distribution of BacP-HA. Cells of strain LL046 (*bacP-HA* P_*parB*_-*parB-eyfp*) were subjected to immunofluorescence microscopy with anti-HA antibodies and treated with DAPI to visualize the nucleoids. The population-wide distribution of the immunofluorescence signals was visualized by demographic analysis (on the left; *n* = 150 cells). An exemplary overlay of the immunofluorescence and DAPI signals is shown on the right (bar: 3 µm). **e** Unequal distribution of chromosomal DNA and ParB·origin complexes in the presence of BacP-HA. Cells of strain LL046 were treated with DAPI and analyzed by fluorescence microscopy (bar: 3 µm). **f** Asymmetric subcellular distribution of ParA-mCherry in the presence of BacP-HA. Cells of strain LL150 (*bacP-HA* P_*parA*_-*parA-mCherry*) were imaged by fluorescence microscopy. The population-wide distribution of the fluorescence signals was visualized by demographic analysis (on the left; *n* = 116 cells). An exemplary fluorescence image is given on the right (bar: 3 µm)

The lack of BacNOP or PadC strongly affects the subcellular arrangement of the ParAB*S* chromosome partitioning machinery. To clarify the physiological consequences, we first determined the dimensions of the nucleoids in various mutant backgrounds. Although bactofilin-deficient strains did not show any appreciable changes in cell length and growth rate (Supplementary Fig. 8a and b), the nucleoids of bactofilin and *padC* mutants were significantly more compact, with their longitudinal sizes decreasing from 51% of the cell length in the wild type to only 37% in the Δ*bacNOP* Δ*padC* strain (Fig. 7a). Apart from this change in nucleoid size, bactofilin mutants often displayed an abnormal chromosome arrangement, with their origin regions displaced from the pole-proximal edges to more central regions of the nucleoids (Fig. 7b). Notably, using ParB-YFP as a label for the chromosomal origin regions, we identified a moderate increase in origin copy numbers in the Δ*bacP* background (Supplementary Fig. 8d). In line with this observation, populations of Δ*bacNOP* cells exhibited a noticeable fraction of cells with abnormally high DNA content (Fig. 7c), suggesting that the proper positioning of ParAB*S* helps to make chromosome segregation more robust.

We fortuitously observed that fusion of BacP with the HA affinity tag created a variant that formed extended unipolar, instead of bipolar, patches (Fig. 7d), providing a means to test the role of bactofilins on ParAB localization in a non-native context. Interestingly, *bacP-HA* cells showed impaired growth (Supplementary Fig. 8c) and a severe chromosome segregation defects, with many of them containing either more than two (17%) or no (8%) ParB-YFP complexes (Supplementary Fig. 8d). Consistently, a large fraction of the population contained an abnormal number of chromosome equivalents (Fig. 7c), resulting in part from divisions over the nucleoid (Fig. 7e). Moreover, even in cells containing two chromosomes, the origin regions were severely mislocalized and, in most cases, located in close proximity rather than at opposite edges of the nucleoid (Fig. 7e). Importantly, in the mutant cells, ParA-mCherry had lost its typical bipolar localization pattern and displayed the same unipolar distribution as BacP-HA (Fig. 7f). The asymmetric positioning of ParA and the concomitant sequestration of multiple chromosomal origin regions to a single bactofilin patch (see also Supplementary Fig. 8e) thus appears to severely impede chromosome segregation. Collectively, these findings strongly support a model in which BacNOP form cytoskeletal structures that control the positioning of the ParAB*S* chromosome segregation machinery within the cell.

## Discussion

Apart from the universally conserved homologs of actin and tubulin, there are several groups of cytoskeletal proteins that are exclusively found in bacteria. Among them are the bactofilins, a widespread and highly conserved group of proteins whose biology is still largely unexplored^4^. In this work, we demonstrate that three bactofilin homologs in *M. xanthus* co-assemble into extended subpolar scaffolds which, together with the newly discovered protein PadC, control the positioning of the chromosomal origin segregation machinery. Unlike other bacterial landmark proteins, these structures do not recruit their interaction partners to the very tips of the cells but to well-defined positions within the cytoplasmic space, located at a considerable distance from the cell poles. The establishment of this additional, subpolar domain expands the range of potential protein localization sites, providing a new mechanism for cellular organization that may facilitate the assembly of multiple large macromolecular complexes within the polar or subpolar regions of the cell.

This study shows that BacN, BacO, and BacP consistently colocalize in the cell, indicating that they assemble into a joint polymeric structure. However, each of the three proteins can form filaments on its own in vitro^41^. Therefore, it remains to be clarified whether the three paralogs polymerize into homopolymeric structures that subsequently assemble into heteromeric complexes or whether they associate randomly into mixed polymers. Notably, the functional contributions of the different paralogs vary significantly. Whereas BacN is largely redundant for the processes analyzed in this study, BacO is important for proper assembly of the bactofilin patches. The most pronounced phenotypes, however, are observed upon inactivation of BacP, which not only plays a central role in the formation of bactofilin patches but also mediates the recruitment of PadC and, thus, ParA to these structures. Apart from the architecture of the BacNOP complex, the precise subcellular location of the polymers formed is still unknown. BacNOP could potentially assemble into cytoplasmic filament bundles. On the other hand, recent work has demonstrated that bactofilins not only form filaments but also extensive two-dimensional arrays in vitro, depending on the experimental conditions^44^. Consistently, live-cell imaging and electron cryo-tomographic studies suggest that the *C. crescentus* homologs assemble into sheet-like structures lining the inner face of the cytoplasmic membrane in vivo^41^. It is, therefore, conceivable that *M. xanthus* BacNOP may form similar membrane-associated assemblies, but clarification of this issue will require the development of fully functional fluorescent protein fusions.

**Fig. 8:**
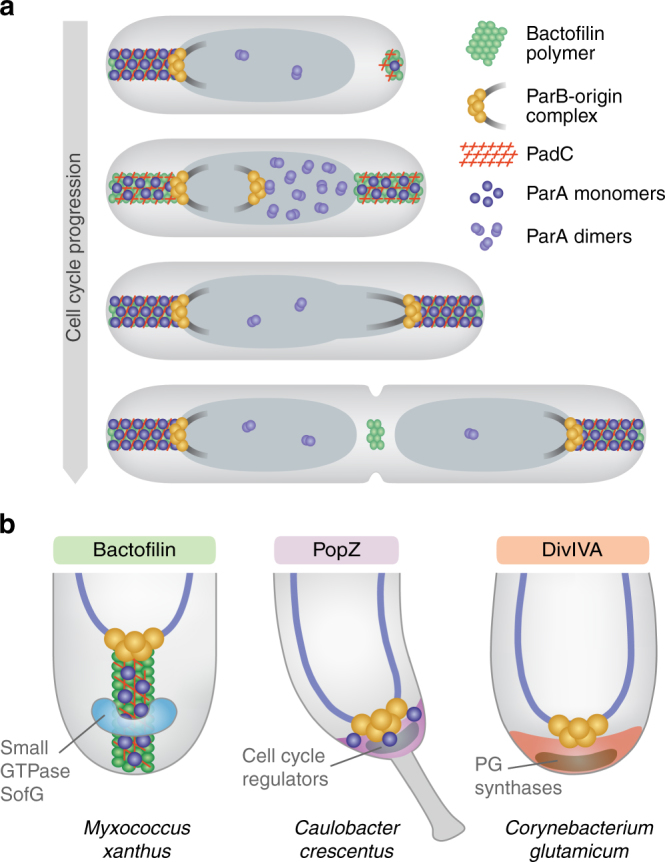
Model for the function of bactofilins in *M. xanthus*. **a** Organization of the *M. xanthus* chromosome segregation machinery by bipolar BacNOP·PadC complexes. Bactofilin structures assemble in a cell cycle-dependent manner. They interact with the adapter protein PadC, which in turn captures ParA monomers and thus mediates their retention in the subpolar regions of the cell. The tips of the bactofilin structures bind to the chromosomal ParB·*parS* complexes, thereby ensuring the proper arrangement of the two sister chromosomes after their segregation by nucleoid-associated ParA dimers. **b** Comparison of the polar scaffolding proteins BacNOP from *M. xanthus*, PopZ from *C. crescentus*, and DivIVA from the actinomycete *C. glutamicum*. Despite their distinct evolutionary origins, all of these proteins function in the organization of the ParAB*S* chromosome segregation machinery. Moreover, they all interact with additional pole-associated factors, serving as multi-purpose hubs that help to spatially organize distinct cellular pathways

Despite the lack of nucleotide cofactors, the BacNOP structures assemble in a tightly controlled and cell cycle-dependent manner. New-born cells often display two differently sized complexes, a longer one at the old pole and a shorter one at the new pole, whose lengths gradually equalize as the cells grow. Before cell division, an additional patch is formed at midcell. Its dissection during cytokinesis then re-establishes a nascent bactofilin complex at the new pole of the daughter cells (Fig. 8a). Notably, cell cycle-regulated localization dynamics have also been observed for the bactofilin clusters of *C. crescentus* ^41^. The mechanisms controlling BacNOP assembly and localization still remain to be determined. However, given that bactofilins polymerize independently of nucleotide cofactors^41, 45^, their assembly may be regulated through protein–protein interactions. Of notice, inactivation of PadC led to a change in the localization pattern of BacNOP (Supplementary Fig. 5e). Apart from recruiting ParA, this protein could therefore also be involved in coordinating bactofilin patch formation with cell cycle events such as chromosome replication or segregation.

We show that BacNOP serve to position the ParAB*S* chromosome segregation machinery within the cell. Interestingly, despite being encoded immediately downstream of the *parAB* genes, their paralog BacM appears not to be involved in this process but to function exclusively in cell shape maintenance^42^. Consistent with this notion, it lacks the typical bipolar localization pattern of BacNOP^41, 42^, supporting the idea that paralogous bactofilins can act independently in distinct cellular pathways. Despite their functional diversity, bactofilins from different species may share a common role as localization factors for other proteins^4^. However, the determinants responsible for the recruitment of interacting factors have remained unknown. Our results now identify the long C-terminal extension of BacP as a central mediator of bactofilin function in *M. xanthus*, serving as a hub for the assembly of the PadC·ParA complex. Intriguingly, the ParA-binding (ParB_C_) domain of PadC bears resemblance to the centromer-binding protein ParB, suggesting that the two proteins may use a similar mode of interaction with their common target ParA. However, structural analyses of the respective complexes are necessary to further investigate this possibility. Whereas our results clarify the pathway of ParA recruitment, the mechanism underlying the immobilization of ParB at the ends of the BacNOP·PadC assemblies is still unclear. We did not observe any binding of purified ParB to the C-terminal extension of BacP or the ParB_C_ domain of PadC in vitro (Supplementary Fig. 2g). The protein may thus interact with the bactofilin core domain or other regions of BacP, BacO, and/or PadC, which are however not amenable to biochemical analysis at this point.

Interestingly, there are striking parallels in the (sub)polar targeting of ParA in *M. xanthus* and *C. crescentus*. Only the monomeric forms of *M. xanthus* ParA are efficiently recruited to the bactofilin·PadC complex in vivo. Dimeric variants, by contrast, localize to the nucleoid or ParB, but they are redirected to the subpolar regions when impaired in DNA binding. Exactly the same pattern was observed for the interaction of ParA with the polar scaffolding protein PopZ in *C. crescentus* ^34, 35^. In this species, the accumulation of ParA monomers within the PopZ matrix was suggested to confine ParA dimerization to the polar regions of the cell, thereby creating a gradient of DNA-bound dimers that dictates the directionality of the segregation process^40^. It is likely that the sequestration of ParA by the bactofilin·PadC complex serves a similar function during chromosome segregation in *M. xanthus*, but the precise mechanistic implications of this phenomenon still remain to be investigated. Interestingly, although ParA and ParB are essential in *M. xanthus* ^50, 51^, inactivation of BacNOP or PadC has only a moderate effect on the overall efficiency of chromosome segregation, at least during vegetative growth. The BacNOP-PadC system may thus have an auxiliary function that optimizes cellular fitness by enhancing the robustness of the segregation process. However, it remains to be clarified whether it may play a more critical role during the formation or outgrowth of myxospores, a feature typical of many species that possess the BacNOP and PadC proteins.

Apart from its role in chromosome organization, BacP has also been implicated in the positioning of a small GTPase, SofG, involved in the regulation of *M. xanthus* motility^49^. BacNOP structures thus serve as multi-purpose scaffolds that interact with factors involved in seemingly unrelated cellular pathways. A similar functional versatility is observed when comparing bactofilin homologs from different bacterial species^4^. It is likely that all bactofilins share the ability to form polymeric structures, based on their conserved DUF583 domain^44^, serving as scaffolds for the assembly and localization of protein complexes. However, the nature of the proteins they recruit appears to vary between systems, resulting in the observed functional diversification.

Intriguingly, there are striking functional analogies between BacNOP and other polar scaffolding proteins such as PopZ and DivIVA, although there is no evolutionary or structural relationship between these factors (Fig. 8b). Similar to BacNOP patches, *C. crescentus* PopZ^22, 23^ and DivIVA homologs from actinomycetes^17^ interact with the centromere-binding protein ParB to control the positioning of the chromosomal origin regions. Moreover, both proteins interact with the chromosome partitioning ATPase ParA. This association can be either direct, as reported for PopZ and DivIVA from *M. smegmatis* ^16, 40^, or mediated through an adapter protein such as the coiled-coil-rich protein Scy in *S. coelicolor* ^18^. Moreover, each of these proteins interacts with additional factors not involved in chromosome segregation. PopZ, for instance, also mediates the polar localization of various proteins involved in *C. crescentus* cell cycle regulation^22, 26^, whereas DivIVA additionally organizes the polar peptidoglycan biosynthetic machinery of actinomycete species^21, 55, 56^. Moreover, DivIVA was shown to recruit another cytoskeletal structure, formed by the intermediate-filament-like protein FilP, to the growing cell poles of *S*. *coelicolor* hyphae^57, 58^. Notably, there are also non-polymerizing proteins that act as multi-functional polar localization factors, including HubP, which mediates the polar recruitment of ParA, the flagellar apparatus, and chemotaxis arrays in *Vibrio cholerae* ^59^. Thus, many bacteria have a common need for pole-organizing factors that help arrange the chromosome segregation machinery and diverse macromolecular complexes within the cell. However, different evolutionary lineages have obviously found very different solutions to cope with this problem.

The reason why *M. xanthus* has evolved a mechanism to position proteins in the subpolar regions and not, as observed for other species, at the very poles of the cell is still unclear. However, a prominent feature of *M. xanthus* is its intricate motility machinery, whose coordination and activity involves an array of pole-associated structural and regulatory proteins^60^. These factors may occupy a large part of the polar cell envelope and thus not leave sufficient space for other large macromolecular structures to assemble at the same site without causing steric or regulatory interference. It will be interesting to see whether other bacterial groups also use bactofilins to establish comparable subpolar domains and, thereby, expand their repertoire of potential protein localization sites.

## Methods

### Media and growth conditions

*M. xanthus* DK1622 and its derivatives were grown at 32 °C in CTT medium^61^, supplemented with kanamycin (50 µg/ml) or oxytetracycline (10 µg/ml) when appropriate. *E. coli* strains were cultivated at 37 °C in LB medium containing antibiotics at the following concentrations (µg/ml in liquid/solid medium): ampicillin (100/200), chloramphenicol (20/30), kanamycin (30/50), tetracycline (15/15), gentamycin (15/20), spectinomycin (50/100). To induce the expression of genes from the P*van*, P*cop*, P*tet* or P*lac* promoters, media were supplemented with sodium vanillate, copper sulfate, anhydrotetracycline (aTet) or isopropyl-β-D-thiogalactopyranoside (IPTG), respectively, as indicated in the text.

### Construction of plasmids and strains

The bacterial strains and plasmids used in this work are described in Supplementary Tables 1–4. The oligonucleotides used for their construction are listed in Supplementary Table 5. All plasmids were verified by DNA sequencing. *M. xanthus* was transformed by electroporation^62^. Non-replicating plasmids were integrated into the *M. xanthus* chromosome by site-specific recombination at the phage Mx8 *attB* site^63^ or by single-homologous recombination at the *cuoA* ^64^ or MXAN_18/19^65^ locus. Gene replacement was achieved by double-homologous recombination using the counter-selectable *galK* marker^66^. Proper chromosomal integration or gene replacement was verified by colony PCR.

### Live-cell imaging

Exponentially growing cells were spotted on pads made of 1.5 % agarose in H_2_O (*E. coli*) or 1.5 % agarose in TPM buffer (10 mM Tris/HCl, 1 mM potassium phosphate, 8 mM MgSO_4_, pH 7.6) (*M. xanthus*). Images were taken with a Zeiss Axio Imager.M1 microscope equipped with a Zeiss Plan Apochromat ×100/1.40 Oil DIC objective and a Cascade:1K CCD camera (Photometrics) or with a Zeiss Axio Imager.Z1 microscope equipped with a ×100/1.46 Oil DIC objective and a pco.edge sCMOS camera (PCO). An X-Cite 120PC metal halide light source (EXFO, Canada) and ET-DAPI, ET-CFP, ET-YFP or ET-TexasRed filter cubes (Chroma, USA) were used for fluorescence detection. Nucleoids were visualized by incubating cells with 0.5 µg/ml 4′,6-diamidino-2-phenylindole (DAPI) for 15–20 min prior to analysis. Images were recorded and processed with Metamorph 7.7 (Molecular Devices).

### Immunofluorescence microscopy

Immunofluorescence microscopy was performed essentially, as described^67^. Cells were grown to exponential phase and fixed with 1.6–2.6 % (w/v) paraformaldehyde and 0.008 % (w/v) glutaraldehyde. After permeabilization in GTE buffer (20 mM Tris/HCl, pH 7.6, 50 mM glucose, 10 mM EDTA), the fixed cells were incubated with suitable antibodies in PBS buffer (137 mM NaCl, 2.7 mM KCl, 10 mM Na_2_HPO_4_, 2 mM KH_2_PO_4_) containing 2 % (w/v) bovine serum albumin (BSA; Carl-Roth, Germany). First, target proteins were labeled with a polyclonal anti-BacO or anti-BacP^49^ antibody or a monoclonal anti-HA antibody (Millipore) at dilutions of 1:500, 1:400, and 1:200, respectively. Immunocomplexes were then visualized with Alexa-Fluor 594 Goat Anti-Rabbit or Alexa-Fluor 488 Goat Anti-Rabbit secondary antibodies (Molecular Probes) at a dilution of 1:200. Before imaging, SlowFade® Antifade (Invitrogen) was applied to each sample.

### Flow cytometry

Cultures were grown to exponential phase, diluted to an OD_550_ of 0.1, and treated for 40 min with the DNA-specific fluorescent dye Vybrant DyeCycle Orange (Invitrogen) at a final concentration of 10 µM. Subsequently, cells were analyzed by flow cytometry in a customized Fortessa Flow Cytometer (BD Biosciences), using an excitation wavelength of 488 nm and a Blue Green 542/27 band-pass emission filter. Data were acquired using FACSdiva 8.0 (BD Biosciences) and processed in FlowJo v10 (FlowJo LLC).

### Growth curves

*M. xanthus* cells were grown to exponential phase, diluted with fresh medium to an OD_550_ of 0.025, and transferred in 24-well polystyrene microtiter plates. Growth was then monitored in an Infinite® M1000 PRO scanner (Tecan) by measuring the optical density at 550 nm (OD_550_) at 15 min intervals, with three replicates per strain. Alternatively, cells were grown in Erlenmeyer flasks, sampled manually at defined intervals, and analyzed in an Ultrospec 2100 pro spectrophotometer (GE Healthcare).

### Protein purification

To purify His_6_-BacP_∆1-115_ (BacP_C_), *E. coli* Rosetta(DE3)pLysS was transformed with plasmid pIB154^49^ and grown at 37 °C in LB medium. At an OD_600_ of 0.6, the cells were induced with 1 mM IPTG and cultivated for another 12 h at 18 °C. They were then harvested by centrifugation, washed twice with buffer B1 (50 mM NaH_2_PO_4_, 300 mM NaCl, 10 mM imidazole, adjusted to pH 8.0 with NaOH), and stored at −80 °C. Thawed cells were resuspended in buffer B2 (50 mM NaH_2_PO_4_, 300 mM NaCl, 10 mM imidazole, 1 mM ß-mercaptoethanol, pH 8.0) containing 10 μg/ml DNase I and 100 μg/ml PMSF and disrupted by three passages through a French press (16,000 psi). After the removal of cell debris by centrifugation for 30 min at 30,000 xg, the cleared lysate was applied to a 5 ml HisTrap HP column (GE Healthcare) equilibrated with buffer B3 (50 mM NaH_2_PO_4_, 300 mM NaCl, 1 mM β-mercaptoethanol, pH 8.0) containing 20 mM imidazole. The column was washed with 5 column volumes (CV) of the same buffer, and protein was eluted with a linear imidazole gradient (20–250 mM in buffer B3) at a flow rate of 2 ml/min. Fractions containing high concentrations of protein were pooled and dialyzed against 3 l of buffer P (25 mM HEPES/KOH, pH 7.6, 100 mM KCl, 10% (v/v) glycerol), The solution was then aliquoted, snap-frozen in liquid N_2_, and stored at −80 °C until further use.

To purify His_6_-PadC_∆1-281_ (PadC_ΔN_), *E. coli* Rosetta(DE3)pLysS was transformed with plasmid pMO002 and grown at 37 °C in LB medium (3 l). At an OD_600_ of 0.8, protein overproduction was induced with 0.5 mM IPTG for 4 h. Cells were harvested, washed with buffer, and resuspended in buffer B2 containing 10 μg/ml DNase I and 100 μg/ml PMSF. After three passages through a French press (16,000 psi), the cell lysate was clarified by centrifugation at 30,000×g for 30 min, and the supernatant was applied onto a 5 ml HisTrap HP column (GE Healthcare) previously equilibrated with buffer B3 containing 20 mM imidazole. The column was washed with 5 CV of the same buffer, and protein was eluted with a linear imidazole gradient (20–250 mM in buffer B3) at a flow rate of 2 ml/min. Fractions containing high concentrations of protein were pooled and dialyzed against 3 l of buffer B5 (20 mM Tris/HCl, pH 8.0, 10 mM NaCl, 1 mM β-mercaptoethanol) at 4 °C. After the removal of precipitates by centrifugation at 30,000×*g* for 30 min, the solution was loaded onto a MonoQ 5/50 column (GE Healthcare) equilibrated with buffer B5. The column was washed with 20 CV of buffer B5 prior the application of a linear NaCl gradient (0.01–1 M NaCl in buffer B5) at a flow rate of 1 ml/min. Fractions containing the purified protein were pooled and dialyzed against 2 l of buffer C7 (25 mM HEPES/KOH, pH 8.0, 20 mM NaCl, 0.1 mM EDTA, 5 mM MgCl_2_, 1 mM β-mercaptoethanol, 10 % (v/v) glycerol), snap-frozen, and stored at −80 ˚C until further use.

His_6_-ParA was purified essentially as described previously^38^. *E. coli* Rosetta(DE3)pLysS cells carrying plasmid pAH17^50^ were grown to an OD_600_ of 0.6 at 37 °C in LB medium (3 l). The cultures were chilled to 18 °C, and 1 mM IPTG was added to induce His_6_-ParA synthesis overnight at 18 °C. Cells were harvested by centrifugation, washed twice with buffer A1 (100 mM HEPES/KOH, pH 7.4, 100 mM KCl, 100 mM EDTA, 10% (v/v) glycerol), and resuspended in 25 ml of buffer A1 containing 10 µg/mL DNase I, 100 µg/mL PMSF, 0.5 mM MgATP, and 1 mM DTT. The cell suspension was incubated on ice for 20 min prior to addition of 4 M KCl to a final concentration of 1 M. Cells were disrupted by three passages through a French press (16,000 psi), and cell debris was removed by centrifugation at 30,000×*g* and 4 °C for 30 min at. The clarified lysate was applied onto a 5 ml HisTrap HP column (GE Healthcare) equilibrated with buffer A2 (25 mM HEPES/KOH, pH 7.4, 450 mM KCl, 50 mM potassium glutamate, 1 mM MgSO_4_, 1 mM DTT, 100 µM magnesium-ATP) containing 40 mM imidazole. After a wash with 5 CV of the same buffer, protein was eluted with a linear imidazole gradient (40–300 mM in buffer A2) at a flow rate of 2 ml/min. Fractions containing high concentrations of His_6_-ParA were pooled, dialyzed against 2 l of buffer A4 (25 mM HEPES/KOH, pH 7.4, 100 mM KCl, 200 mM potassium glutamate, 1 mM MgSO_4_, 1 mM DTT, 100 µM magnesium-ATP, 20% (v/v) glycerol), snap-frozen, and stored at −80 °C until further use.

BacO-His_6_ was produced and purified as described previously^41^.

To purify StrepII-ParB, *E. coli* Rosetta(DE3)pLysS was transformed with pLL80 and grown at 37 °C in 500 ml of LB medium. At an OD_600_ of 1, expression was induced with 0.5 mM IPTG for 3 h. Cells were harvested, washed twice with buffer B1, and resuspended in buffer NP (50 mM NaH_2_PO_4_, 300 mM NaCl, adjusted to pH 8.0 with NaOH) containing 10 μg/ml DNase I and 100 μg/ml PMSF. After three passages of the cells through a French press (16,000 psi), the lysates were cleared by centrifugation at 30,000×g for 30 min, mixed with Strep-Tactin^®^ Superflow Plus (Qiagen) resin, and incubated with gentle agitation for 2 h at 4 °C. The resin was washed three times with buffer NP, and proteins were eluted with buffer NPD (50 mM NaH_2_PO_4_, 300 mM NaCl, 2.5 mM dethiobiotin, adjusted to pH 8.0 with NaOH). The eluate was dialyzed against 2.5 l of dialysis buffer (50 mM NaH_2_PO_4_, 150 mM NaCl, 1 mM EDTA, adjusted to pH 8.0 with NaOH), snap-frozen, and stored at −80 ˚C until further use.

To purify His_6_-PadC_∆1-239_, *E. coli* Rosetta(DE3)pLysS was transformed with pLL105 and grown at 37 °C in 750 ml of LB medium. At an OD_600_ of 0.8, expression was induced with 0.5 mM IPTG for 4 h. Cells were harvested, washed with buffer B1, and resuspended in buffer B2 containing 10 μg/ml Dnase I and 100 μg/ml PMSF. After three passages through a French press (16,000 psi), cell debris was removed by centrifugation at 30,000×g for 30 min. The cleared lysates were then mixed with Ni-NTA agarose beads (Qiagen) that had been equilibrated with buffer B2 for 2 h at 4 ˚C. The beads were washed with buffer B3 containing 20 mM imidazole, and protein was eluted with buffer B3 containing 250 mM imidazole. The eluate was dialyzed against 3 l of buffer B6 (50 mM HEPES, pH 7.2, 50 mM NaCl, 5 mM MgCl_2_, 0.1 mM EDTA, 10% (v/v) glycerol, 1 mM ß-mercaptoethanol), snap-frozen, and stored at −80 ˚C until further use.

### Antibodies and immunoblot analysis

Polyclonal anti‐BacO and anti-PadC antibodies were raised by immunization of rabbits with purified BacO-His_6_ or His_6_-PadC_Δ1-239_ (Eurogentec). Immunoblot analysis was performed as described previously^30^, using a polyclonal anti-BacO, anti-BacP^49^, anti-PadC, or anti-ParB^50^ antibody or a monoclonal anti-HA antibody (Millipore) at dilutions of 1:7500 (anti-BacO), 1:1000 (anti-BacP), 1:2500 (anti-PadC), 1:5000 (anti-ParB), or 1:8000 (anti-HA).

### Bio-layer interferometry

Bio-layer interferometry experiments were conducted using a BLItz system equipped with High Precision Streptavidin (SAX) Biosensors (ForteBio). BacP_C_ and PadC_∆N_ were biotinylated with EZ-Link NHS-PEG4-Biotin (Thermo Scientific) as recommended by the manufacturer. After immobilization of the biotinylated proteins on the sensors and establishment of a stable baseline, association reactions were monitored at various analyte concentrations. At the end of each binding step, the sensor was transferred into analyte-free buffer to follow the dissociation kinetics. The extent of non-specific binding was assessed by monitoring the interaction of analyte with unmodified sensors. All analyses were performed in BLItz binding buffer (25 mM HEPES/KOH, pH 7.6, 100 mM KCl, 10 mM MgSO_4_, 1 mM DTT, 10 µM BSA, 0.01% Tween). Reactions involving ParA were additionally supplemented with 150 mM potassium glutamate, 5% glycerol, and 10 mM ATP.

### Co-purification analysis

To identify interaction partners of BacN-HA, exponentially growing cultures (500 ml) of strains DK1622 and LL033 were treated for 20 min at 37 °C with 0.6% paraformaldehyde in PBS (pH 8.0). The cross-linking reaction was stopped by addition of 125 mM glycine in PBS (pH 8.0), and the culture was harvested by centrifugation at 12,000 g for 20 min at 4 °C. After three washes with 200 ml PBS (pH 8.0), the cells were resuspended in 6 mL of Co-IP buffer (50 mM Tris/HCl, pH 7.6, 150 mM NaCl, 0.1% Triton X-100) supplemented with Complete Mini EDTA-free protease inhibitor (Roche) and disrupted by three passages through a French press (16,000 psi). The suspension was clarified by centrifugation at 12,000×*g* for 10 min at 4 °C, and the supernatant was incubated with anti-HA-tag mAb Magnetic Beads (MBL Life science) for 12 h at 4 °C. The beads were then washed three times with 1.5 ml of Co-IP buffer, resuspended in SDS sample buffer, and incubated for 20 min at 99 °C to elute bound protein. Samples were taken at different steps of the procedure and subjected to immunoblot analysis using anti-HA, anti-BacP and anti-BacO antibodies.

To identify interaction partners of StrepII-ParB, an exponentially growing culture (1 l) of wild-type strain DK1622 was treated with 0.6% paraformaldehyde in PBS. The cross-linking reaction was stopped by addition of 125 mM glycine in PBS. Cells were harvested, resuspended in 15 ml buffer S (20 mM Tris-HCl, pH 7.6, 200 mM NaCl) supplemented with Complete Mini with EDTA protease inhibitor (Roche), and lysed by three passages through a French press (16,000 psi). After the removal of cell debris, the cleared lysate was mixed with Strep-Tactin® Superflow Plus resin that had been pre-incubated with 1 mg purified StrepII-ParB in buffer S. A similar mixture with beads not coupled to purified protein served as a negative control. After incubation overnight at 4 °C, the beads were washed with buffer S, and protein was eluted with NPD buffer (50 mM NaH_2_PO_4_, 300 mM NaCl, 2.5 mM dethiobiotin, adjusted to pH 8.0 with NaOH). The eluates were then concentrated with trichloroacetic acid and probed with anti-BacP antibodies.

To identify interaction partners of His_6_-PadC_∆1-239_, cells of wild-type strain DK1622 (2 l) were treated with paraformaldehyde, harvested, and washed as described for StrepII-ParB. The cells were resuspended in 20 ml buffer S supplemented with Complete Mini without EDTA protease inhibitor (Roche) and lysed by three passages through a French press (16,000 psi). After the removal of cell debris, the cleared lysate was mixed with Ni-NTA agarose (Qiagen) beads that had been pre-incubated for 1.5 h in buffer S with 1.5 mg purified His_6_-PadC_∆1-239_. A similar mixture containing beads not coupled to purified protein served as negative control. After incubation overnight at 4 °C, the beads were washed with buffer S, and protein was eluted with buffer B3 (50 mM NaH_2_PO_4_, 300 mM NaCl, 1 mM ß-mercaptoethanol, adjusted to pH 8.0 with NaOH) containing 250 mM imidazole. The eluate was then subjected to immunoblot analysis with anti-BacP or anti-BacO antibodies.

### Statistical and bioinformatic analysis

Data were plotted using Origin 6.1 (OriginLab) and QtiPlot 0.9.8.7 (http://www.qtiplot.com/). *t-*tests and Mann–Whitney rank sum tests were performed in SigmaPlot 13 (Systat Software), assuming two independent populations with a significance level of *p* = 0.001. To generate demographs, fluorescence intensity profiles were measured with ImageJ 1.47 v (http://imagej.nih.gov/ij). The data were then processed in R version 3.0.2 (The R Foundation for Statistical Computing; http://www.r-project.org) using the Cell Profiles script (http://github.com/ta-cameron/Cell-Profiles)^68^. Pearson’s correlation coefficients were determined using the Coloc 2 plugin for ImageJ (https://imagej.net/Coloc_2). Nucleotide and amino acid sequences and information on the domain structure of proteins were obtained from the National Center for Biotechnology Information (NCBI) (http://www.ncbi.nlm.nih. gov)^69^. Protein secondary structures were predicted using the PSIPRED Protein Sequence Analysis Workbench (http://bioinf.cs.ucl.ac.uk/psipred)^70^. The prediction of helix-turn-helix motifs was performed at the Pole BioInformatique Lyonnais (https://prabi.ibcp.fr)^71^. The domain structure of proteins was analyzed using the Pfam server^72^.

### Data availability

The authors declare that all relevant data supporting the conclusions of this study are included in the published article and the accompanying Supplementary Information files. Any additional information is available from the corresponding author upon request.

## Electronic supplementary material

Supplementary Information

## References

[1] Rudner DZ, Losick R (2010). Protein subcellular localization in bacteria. Cold Spring Harbor Perspect. Biol..

[2] Shapiro L, McAdams HH, Losick R (2009). Why and how bacteria localize proteins. Science.

[3] Schlimpert S (2012). General protein diffusion barriers create compartments within bacterial cells. Cell.

[4] Lin L, Thanbichler M (2013). Nucleotide-independent cytoskeletal scaffolds in bacteria. Cytoskeleton.

[5] Treuner-Lange A, Søgaard-Andersen L (2014). Regulation of cell polarity in bacteria. J. Cell Biol..

[6] Laloux G, Jacobs-Wagner C (2014). How do bacteria localize proteins to the cell pole?. J. Cell Sci..

[7] Stahlberg H (2004). Oligomeric structure of the *Bacillus subtilis* cell division protein DivIVA determined by transmission electron microscopy. Mol. Microbiol..

[8] Oliva MA (2010). Features critical for membrane binding revealed by DivIVA crystal structure. EMBO J..

[9] Ramamurthi KS, Losick R (2009). Negative membrane curvature as a cue for subcellular localization of a bacterial protein. Proc. Natl Acad. Sci. USA.

[10] Eswaramoorthy P (2011). Cellular architecture mediates DivIVA ultrastructure and regulates min activity in *Bacillus subtilis*. mBio.

[11] Lenarcic R (2009). Localisation of DivIVA by targeting to negatively curved membranes. EMBO J..

[12] Bramkamp M (2008). A novel component of the division-site selection system of *Bacillus subtilis* and a new mode of action for the division inhibitor MinCD. Mol. Microbiol..

[13] Patrick JE, Kearns DB (2008). MinJ (YvjD) is a topological determinant of cell division in *Bacillus subtilis*. Mol. Microbiol..

[14] Ben-Yehuda S, Rudner DZ, Losick R (2003). RacA, a bacterial protein that anchors chromosomes to the cell poles. Science.

[15] Wu LJ, Errington J (2003). RacA and the Soj-Spo0J system combine to effect polar chromosome segregation in sporulating *Bacillus subtilis*. Mol. Microbiol..

[16] Ginda K (2013). ParA of *Mycobacterium smegmatis* co-ordinates chromosome segregation with the cell cycle and interacts with the polar growth determinant DivIVA. Mol. Microbiol..

[17] Donovan C, Sieger B, Kramer R, Bramkamp M (2012). A synthetic *Escherichia coli* system identifies a conserved origin tethering factor in Actinobacteria. Mol. Microbiol..

[18] Ditkowski. B (2013). Dynamic interplay of ParA with the polarity protein, Scy, coordinates the growth with chromosome segregation in *Streptomyces coelicolor*. Open Biol..

[19] Hempel AM, Wang SB, Letek M, Gil JA, Flärdh K (2008). Assemblies of DivIVA mark sites for hyphal branching and can establish new zones of cell wall growth in *Streptomyces coelicolor*. J. Bacteriol..

[20] Letek M (2008). DivIVA is required for polar growth in the MreB-lacking rod-shaped actinomycete *Corynebacterium glutamicum*. J. Bacteriol..

[21] Sieger B, Schubert K, Donovan C, Bramkamp M (2013). The lipid II flippase RodA determines morphology and growth in *Corynebacterium glutamicum*. Mol. Microbiol..

[22] Ebersbach G, Briegel A, Jensen GJ, Jacobs-Wagner C (2008). A self-associating protein critical for chromosome attachment, division, and polar organization in *Caulobacter*. Cell.

[23] Bowman GR (2008). A polymeric protein anchors the chromosomal origin/ParB complex at a bacterial cell pole. Cell.

[24] Bowman GR (2013). Oligomerization and higher-order assembly contribute to sub-cellular localization of a bacterial scaffold. Mol. Microbiol..

[25] Laloux G, Jacobs-Wagner C (2013). Spatiotemporal control of PopZ localization through cell cycle-coupled multimerization. J. Cell. Biol..

[26] Bowman GR (2010). *Caulobacter* PopZ forms a polar subdomain dictating sequential changes in pole composition and function. Mol. Microbiol..

[27] Gerdes K, Howard M, Szardenings F (2010). Pushing and pulling in prokaryotic DNA segregation. Cell.

[28] Wang X, Montero Llopis P, Rudner DZ (2013). Organization and segregation of bacterial chromosomes. Nat. Rev. Genet..

[29] Mohl DA, Gober JW (1997). Cell cycle-dependent polar localization of chromosome partitioning proteins in *Caulobacter crescentus*. Cell.

[30] Thanbichler M, Shapiro L (2006). MipZ, a spatial regulator coordinating chromosome segregation with cell division in *Caulobacter*. Cell.

[31] Toro E, Hong SH, McAdams HH, Shapiro L (2008). *Caulobacter* requires a dedicated mechanism to initiate chromosome segregation. Proc. Natl Acad. Sci. USA.

[32] Shebelut CW, Guberman JM, van Teeffelen S, Yakhnina AA, Gitai Z (2010). *Caulobacter* chromosome segregation is an ordered multistep process. Proc. Natl Acad. Sci. USA.

[33] Leonard TA, Butler PJ, Löwe J (2005). Bacterial chromosome segregation: structure and DNA binding of the Soj dimer – a conserved biological switch. EMBO J..

[34] Schofield WB, Lim HC, Jacobs-Wagner C (2010). Cell cycle coordination and regulation of bacterial chromosome segregation dynamics by polarly localized proteins. EMBO J..

[35] Ptacin JL (2010). A spindle-like apparatus guides bacterial chromosome segregation. Nat. Cell Biol..

[36] Vecchiarelli AG, Hwang LC, Mizuuchi K (2013). Cell-free study of F plasmid partition provides evidence for cargo transport by a diffusion-ratchet mechanism. Proc. Natl Acad. Sci. USA.

[37] Hwang LC (2013). ParA-mediated plasmid partition driven by protein pattern self-organization. EMBO J..

[38] Lim HC (2014). Evidence for a DNA-relay mechanism in ParABS-mediated chromosome segregation. eLife.

[39] Fogel MA, Waldor MK (2006). A dynamic, mitotic-like mechanism for bacterial chromosome segregation. Genes Dev..

[40] Ptacin JL (2014). Bacterial scaffold directs pole-specific centromere segregation. Proc. Natl Acad. Sci. USA.

[41] Kühn J (2010). Bactofilins, a ubiquitous class of cytoskeletal proteins mediating polar localization of a cell wall synthase in *Caulobacter crescentus*. EMBO J..

[42] Koch MK, McHugh CA, Hoiczyk E (2011). BacM, an N-terminally processed bactofilin of *Myxococcus xanthus*, is crucial for proper cell shape. Mol. Microbiol..

[43] Shi. C (2015). Atomic-resolution structure of cytoskeletal bactofilin by solid-state NMR. Sci. Adv..

[44] Vasa S (2015). beta-Helical architecture of cytoskeletal bactofilin filaments revealed by solid-state NMR. Proc. Natl Acad. Sci. USA.

[45] Zuckerman DM (2015). The bactofilin cytoskeleton protein BacM of *Myxococcus xanthus* Forms an extended β-sheet structure likely mediated by hydrophobic interactions. PLoS ONE.

[46] Kassem MM, Wang Y, Boomsma W, Lindorff-Larsen K (2016). Structure of the bacterial cytoskeleton protein bactofilin by NMR chemical shifts and sequence variation. Biophys. J..

[47] Sycuro LK (2010). Peptidoglycan crosslinking relaxation promotes *Helicobacter pylori*’s helical shape and stomach colonization. Cell.

[48] El Andari J, Altegoer F, Bange G, Graumann PL (2015). *Bacillus subtilis* bactofilins are essential for flagellar hook- and filament assembly and dynamically localize into structures of less than 100 nm diameter underneath the cell membrane. PLoS ONE.

[49] Bulyha I (2013). Two small GTPases act in concert with the bactofilin cytoskeleton to regulate dynamic bacterial cell polarity. Dev. Cell.

[50] Harms A, Treuner-Lange A, Schumacher D, Søgaard-Andersen L (2013). Tracking of chromosome and replisome dynamics in *Myxococcus xanthus* reveals a novel chromosome arrangement. PLoS. Genet..

[51] Iniesta AA (2014). ParABS system in chromosome partitioning in the bacterium *Myxococcus xanthus*. PLoS ONE.

[52] Hester CM, Lutkenhaus J (2007). Soj (ParA) DNA binding is mediated by conserved arginines and is essential for plasmid segregation. Proc. Natl Acad. Sci. USA.

[53] Kiekebusch D, Thanbichler M (2014). Spatiotemporal organization of microbial cells by protein concentration gradients. Trends Microbiol..

[54] Vecchiarelli AG (2013). Dissection of the ATPase active site of P1 ParA reveals multiple active forms essential for plasmid partition. J. Biol. Chem..

[55] Meniche X (2014). Subpolar addition of new cell wall is directed by DivIVA in mycobacteria. Proc. Natl Acad. Sci. USA.

[56] Flärdh K (2003). Essential role of DivIVA in polar growth and morphogenesis in *Streptomyces coelicolor* A3(2). Mol. Microbiol..

[57] Fuchino K (2013). Dynamic gradients of an intermediate filament-like cytoskeleton are recruited by a polarity landmark during apical growth. Proc. Natl Acad. Sci. USA.

[58] Holmes NA (2013). Coiled-coil protein Scy is a key component of a multiprotein assembly controlling polarized growth in *Streptomyces*. Proc. Natl Acad. Sci. USA.

[59] Yamaichi Y (2012). A multidomain hub anchors the chromosome segregation and chemotactic machinery to the bacterial pole. Genes Dev..

[60] Zhang Y, Ducret A, Shaevitz J, Mignot T (2012). From individual cell motility to collective behaviors: insights from a prokaryote*. Myxococcus xanthus*. FEMS Microbiol. Rev..

[61] Hodgkin J, Kaiser D (1977). Cell-to-cell stimulation of movement in nonmotile mutants of *Myxococcus*. Proc. Natl Acad. Sci. USA.

[62] Kashefi K, Hartzell PL (1995). Genetic suppression and phenotypic masking of a *Myxococcus xanthus frzF*^-^ defect. Mol. Microbiol..

[63] Magrini V, Creighton C, Youderian P (1999). Site-specific recombination of temperate *Myxococcus xanthus* phage Mx8: genetic elements required for integration. J. Bacteriol..

[64] Gomez-Santos N (2012). Comprehensive set of integrative plasmid vectors for copper-inducible gene expression in *Myxococcus xanthus*. Appl. Environ. Microbiol..

[65] Iniesta AA, Garcia-Heras F, Abellon-Ruiz J, Gallego-Garcia A, Elias-Arnanz M (2012). Two systems for conditional gene expression in *Myxococcus xanthus* inducible by isopropyl-beta-D-thiogalactopyranoside or vanillate. J. Bacteriol..

[66] Ueki T, Inouye S, Inouye M (1996). Positive-negative KG cassettes for construction of multi-gene deletions using a single drug marker. Gene.

[67] Bulyha I (2009). Regulation of the type IV pili molecular machine by dynamic localization of two motor proteins. Mol. Microbiol..

[68] Cameron TA, Anderson-Furgeson J, Zupan JR, Zik JJ, Zambryski PC (2014). Peptidoglycan synthesis machinery in *Agrobacterium tumefaciens* during unipolar growth and cell division. mBio.

[69] NCBI Resource Coordinators. (2016). Database resources of the National Center for Biotechnology Information. Nucleic Acids Res..

[70] Jones DT (1999). Protein secondary structure prediction based on position-specific scoring matrices. J. Mol. Biol..

[71] Dodd IB, Egan JB (1990). Improved detection of helix-turn-helix DNA-binding motifs in protein sequences. Nucleic Acids Res..

[72] Finn RD (2016). The Pfam protein families database: towards a more sustainable future. Nucleic Acids Res..

