# Supplementary material for: Bactofilin-mediated organization of the ParABS chromosome segregation system in Myxococcus xanthus
Source: Nat Commun. 2017 Nov 28;8:1817. doi: 10.1038/s41467-017-02015-z (PMC5703909; doi:10.1038/s41467-017-02015-z)
Supplement: Supplementary file 1 — Supplementary Information [file 41467_2017_2015_MOESM1_ESM.pdf]

SUPPLEMENTARY FIGURES

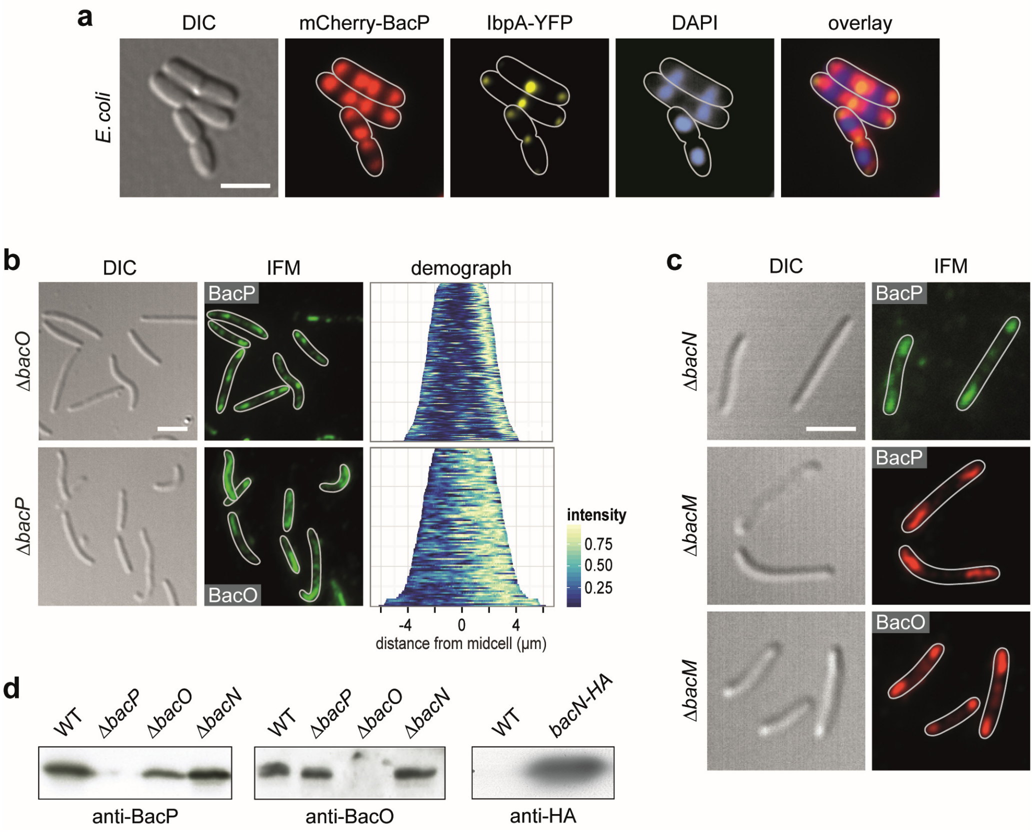

Supplementary Figure 1. Localization and stability of BacP, BacO, and BacN. (a) Differential localization of the bactofilin complexes and inclusion bodies in *E. coli*. Strain BL21(DE3) bearing plasmids pLL54 (P_{T7}-*mCherry-bacP* e*cfp-bacO*) and pLL235 (P_{T7}-*ibpA-eyfp*) was induced with 0.5 mM IPTG (for 1 h) and treated with 30 µg/ml chloramphenicol (for 30 min). After the staining of DNA with DAPI, cells were analyzed by differential interference contrast (DIC) and fluorescence microscopy (bar: 3 µm). Note that the chaperone IbpA is specifically associated with protein aggregates and thus serves as a marker for the subcellular position of inclusion bodies in *E. coli*[1]. **(b)** Interdependence of BacP and BacO localization. Cells of strains LL002 (Δ*bacO*) and LL001 (Δ*bacP*) were analyzed by immunofluorescence microscopy using anti-BacP or anti-BacO antibodies, respectively. An Alexa-Fluor 488-conjugated secondary antibody was used to detect the immunocomplexes (bar: 3 µm). The panels on the right show demographs visualizing the subcellular distributions of BacP and BacO as a function of cell length (n = 172 cells for LL002 and 99 cells for LL001). **(c)** Localization of BacP and BacO in the absence of other bactofilin homologs. Strains LL003 (Δ*bacN*) and MT300 (Δ*bacM*) were analyzed by immunofluorescence microscopy using anti-BacP and/or anti-BacO antibodies, respectively (bar: 3 µm). **(d)** Immunoblot analysis of BacP, BacO, and BacN-HA accumulation in different genetic backgrounds. Strains DK1622 (WT), LL001 (Δ*bacP*), LL002 (Δ*bacO*), LL003 (Δ*bacN*), and LL033 (*bacN-HA*) were subjected to immunoblot analysis with anti-BacP, anti-BacO, or anti-HA antibodies, respectively. Full blots are shown in Supplementary Figure 11.

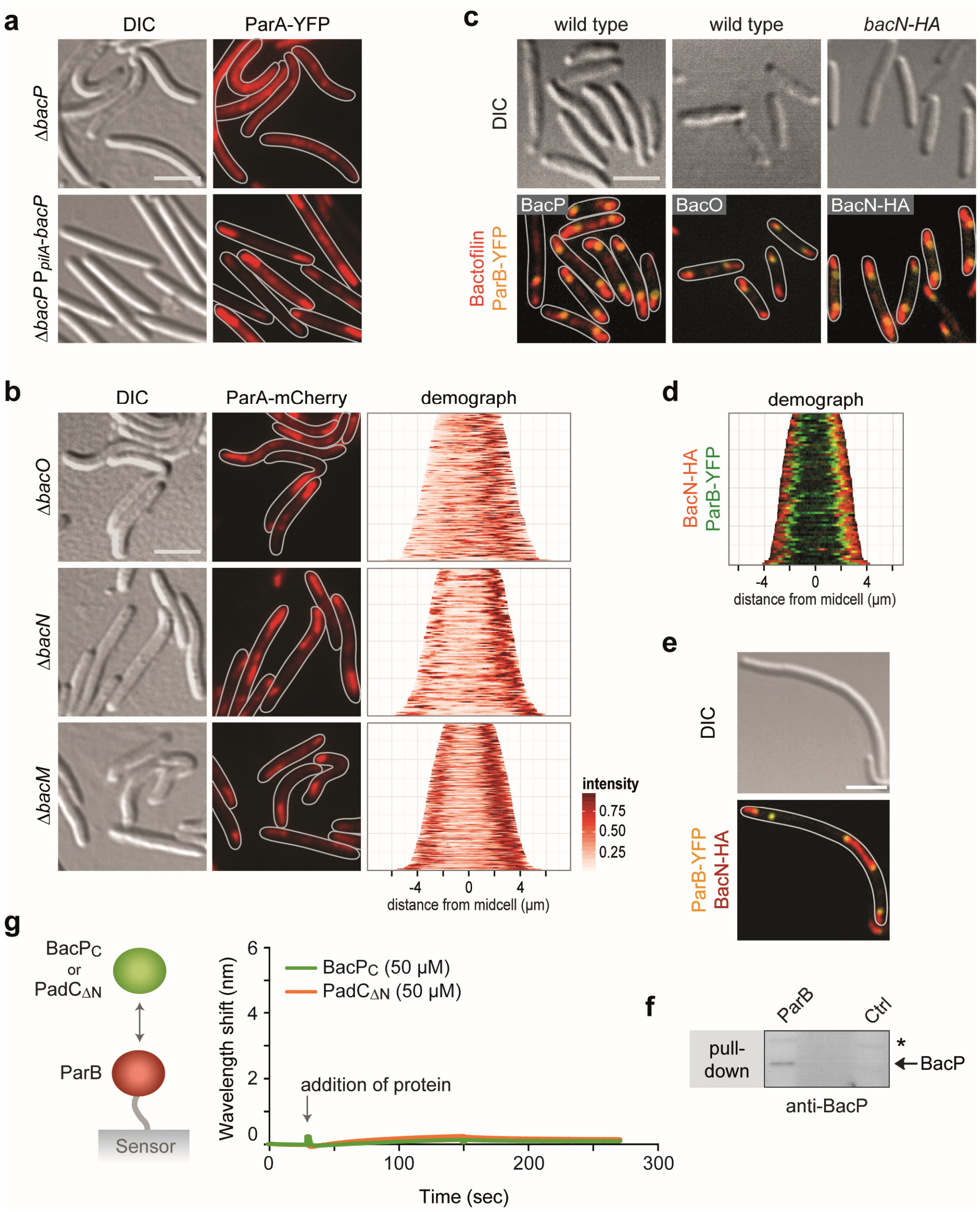

Supplementary Figure 2. BacNOP are important for the subpolar localization of ParA and ParB in *M. xanthus*. (a) Restoration of proper ParA localization by complementation of the *bacP* mutation. Cells of strains LL186 (Δ*bacP* P$_{van}$-*parA-eyfp*) and LL188 (Δ*bacP* P$_{van}$-*parA-eyfp* P$_{pilA}$-*bacP*) were grown in CTT medium, diluted with CTT supplemented with 5 µM vanillate to an OD_{550} of 0.1, and then further incubated for 2 h before imaging (bar: 3 µm). For visualization, the fluorescence signal was false-colored in red. **(b)** Localization of ParA in the absence of BacO, BacN, or BacM. Cells of strains LL148 (Δ*bacO* P$_{parA}$-*parA-mCherry*), and LL149 (Δ*bacN* P$_{parA}$-*parA-mCherry*), and LL151 (Δ*bacM* P$_{parA}$-*parA-mCherry*) were analyzed by DIC and fluorescence microscopy. Demographs summarizing the single-cell fluorescence profiles observed for the three strains are given on the right (n = 162 cells for LL148, 112 cells for LL149, and 234 cells for LL151). **(c)** Colocalization of ParB with polar bactofilin patches. Cells of strains LL012 (P$_{parB}$-*parB-eyfp*) and LL040 (*bacN-HA* P$_{parB}$-*parB-eyfp*) were analyzed by immunofluorescence microscopy, using anti-BacP, anti-BacO (LL012), or anti-HA (LL040) antibodies. The resulting images were overlaid with the ParB-YFP fluorescence signals (bar: 3 µm). **(d)** Localization of ParB to the ends of bactofilin patches. Cells of strain LL040 (*bacN-HA* P$_{parB}$-*parB-eyfp*) were analyzed as described for panel C. Fluorescence profiles showing the subcellular distribution of BacN-HA and ParB-YFP were then arranged according to cell length in a demograph (n = 63 cells). **(e)** Colocalization of ParB with bactofilin structures in filamentous cells. Strain LL040 (*bacN-HA* P$_{parB}$-*parB-eyfp*) was treated for 8 h with 100 µM cephalexin to block cell division and then subjected to immunofluorescence microscopy with an anti-HA primary antibody and an Alexa-Fluor 594-conjugated secondary antibody. Shown are a DIC image and an overlay of the Alexa Fluor 594 and ParB-YFP signals (bar: 3 µm). **(f)** Co-purification of BacP with ParB. A whole-cell lysate of wild-type strain DK1622 was incubated with beads coupled to purified StrepII-ParB. After isolation of the beads, bound protein was eluted, concentrated with trichloroacetic acid, and subjected to immunoblot analysis with anti-BacP antibodies. A reaction performed with beads not pre-incubated with StrepII-ParB served as a control. The full blot is shown in Supplementary Figure 12. **(g)** Bio-layer interferometric analysis showing the absence of a direct interaction between ParB and BacP or PadC *in vitro*. Sensors carrying purified ParB (an amount equivalent to a 3 nm wavelength shift) were probed with 50 µM BacP$_C$ or PadC$_{ΔN}$.

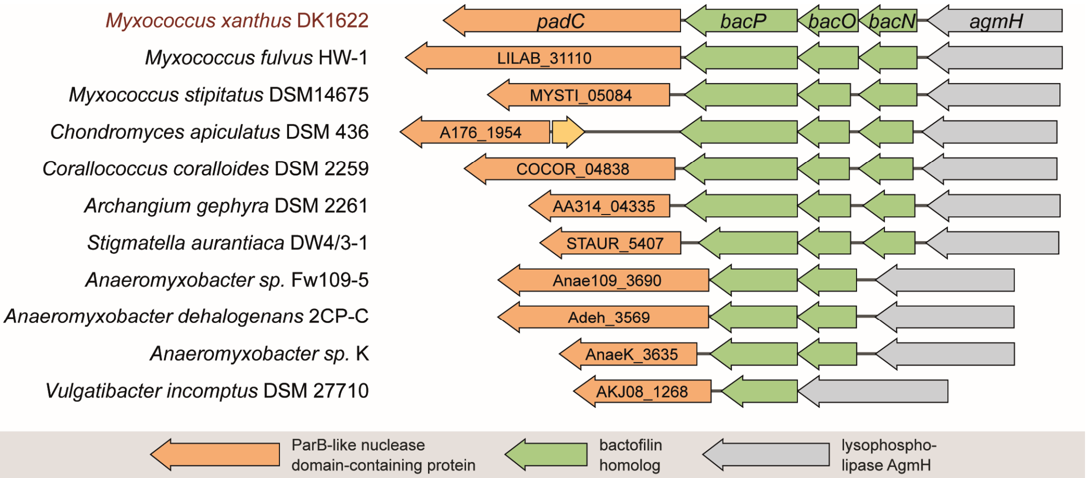

Supplementary Figure 3. Conservation of the *bacP* gene neighborhood in the delta-proteobacterial suborder *Cystobacterineae*. Shown is a comparison of the organization of the bactofilin gene cluster and its neighborhood in representative species of the *Cystobacterineae*. The data were obtained from the SyntTax server[2], using the sequence of *bacP* from *M. xanthus* DK1622 as a query.

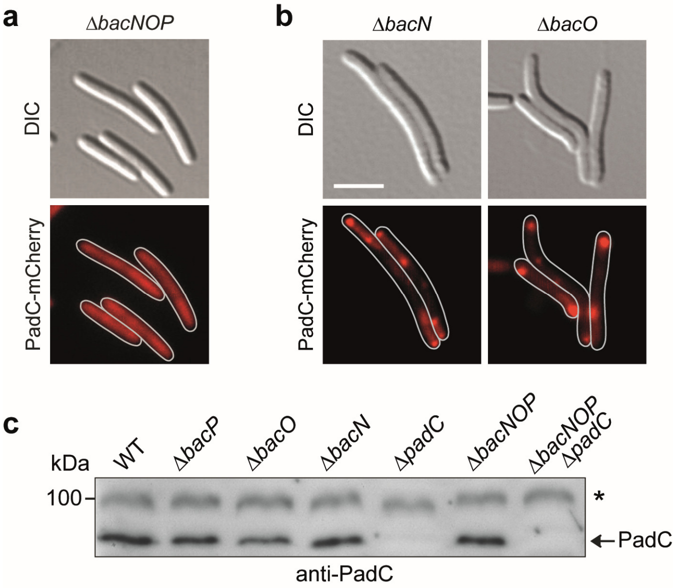

Supplementary Figure 4. PadC is required for the bactofilin-mediated positioning of ParA and ParB. **(a)** Critical role of bactofilins in PadC localization. Cells of strain LL117 (Δ*bacNOP padC-mCherry*) were visualized by DIC and fluorescence microscopy (bar: 3 µm). **(b)** Localization of PadC in the absence of BacN or BacO. Strains LL142 (Δ*bacN padC-mCherry*) and LL141 (Δ*bacO padC-mCherry*) were analyzed by DIC and fluorescence microscopy (bar: 3 µm). **(c)** Levels of PadC in different mutant backgrounds. Cells of strains DK1622 (WT), LL001 *(*Δ*bacP*), LL002 *(*Δ*bacO*), LL003 *(*Δ*bacN*), LL101 *(*Δ*padC*), MT295 *(*Δ*bacNOP*), and LL174 *(*Δ*bacNOP* Δ*padC*) were subjected immunoblot analysis with anti-PadC antibodies. A non-specific cross-reaction with a protein of ~100 kDa (*) serves as a loading control. The full blot is shown in Supplementary Figure 13.

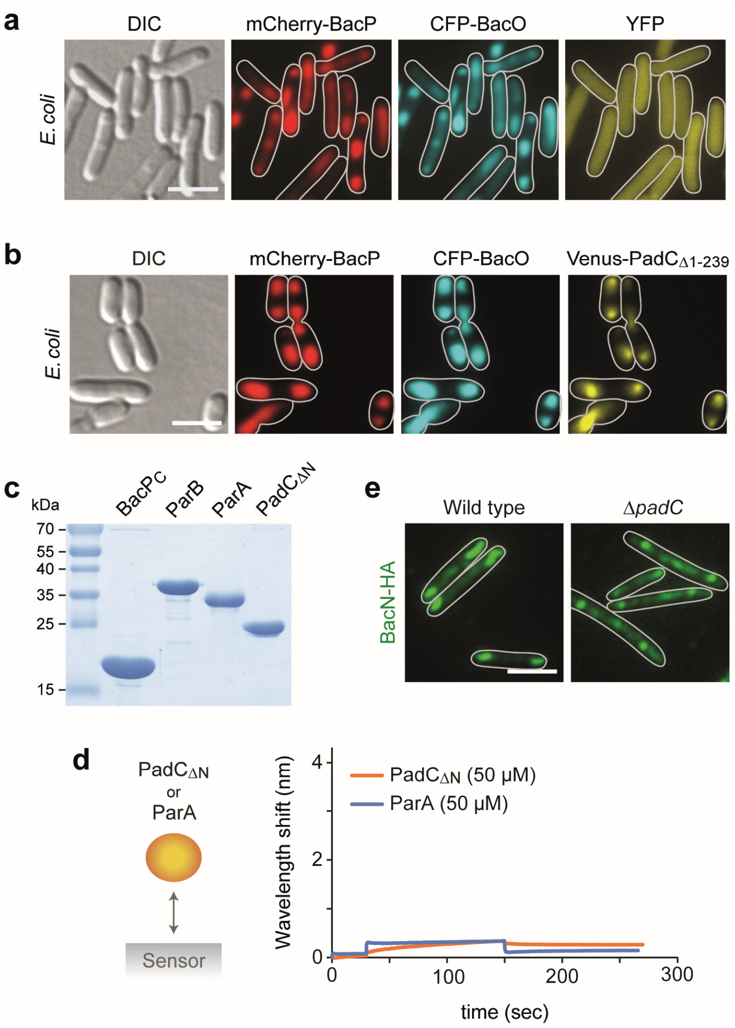

Supplementary Figure 5. PadC is required for the bactofilin-mediated positioning of ParA and ParB. **(a)** Diffuse localization of YFP in *E. coli* cells accumulating bactofilin polymers. Cells of *E. coli* Rosetta(DE3)pLysS bearing plasmids pLL54 (P_{T7}-*mCherry-bacP cfp-bacO*) and pLL61 (P_{tet}-*eyfp*) were induced with 0.5 mM IPTG (for 3.5 h) and 0.2 µg/ml aTet (for 2.5 h) before imaging (bar: 3 µm). The PCC for the mCherry-BacP and YFP signals is 0.45 ± 0.18 (n = 166). **(b)** Co-localization of a PadC derivative lacking the non-structured N-terminal region ($PadC_{\Delta 1-239}$) with bactofilin polymers in *E. coli*. Cells of *E. coli* BL21(DE3) bearing plasmids pLL54 (P_{T7}-*mCherry-bacP cfp-bacO*) and pLL120 (P_{T7}-*venus-padC*$_{\Delta 1-239}$) were induced for 3 h with 0.5 mM IPTG before imaging (bar: 3 µm). The PCC for the mCherry-BacP and Venus-$PadC_{\Delta 1-239}$ signals is 0.89 ± 0.05, n = 61). **(c)** Purity of the proteins used for the bio-layer interferometric analyses. The indicated proteins (10 µg per lane) were separated by SDS gel electrophoresis and stained with Coomassie brilliant blue. A mixture of standard proteins was applied in the rightmost lane. The corresponding molecular weights are indicated next to the gel. **(d)** Lack of interaction between ParA or PadC with unmodified bio-layer interferometry sensors. Sensors lacking any immobilized protein were probed with 50 µM $PadC_{\Delta 1-281}$ or ParA, respectively. **(e)** Aberrant organization of bactofilin structures in the absence of PadC. Cells of strain LL110 (*ΔpadC bacN-HA*) were analyzed by immunofluorescence microscopy using an anti-HA primary antibody and an Alexa-Fluor 488-conjugated secondary antibody. The fluorescence signals obtained are overlaid with the outlines of the cells generated on the basis of DIC images.

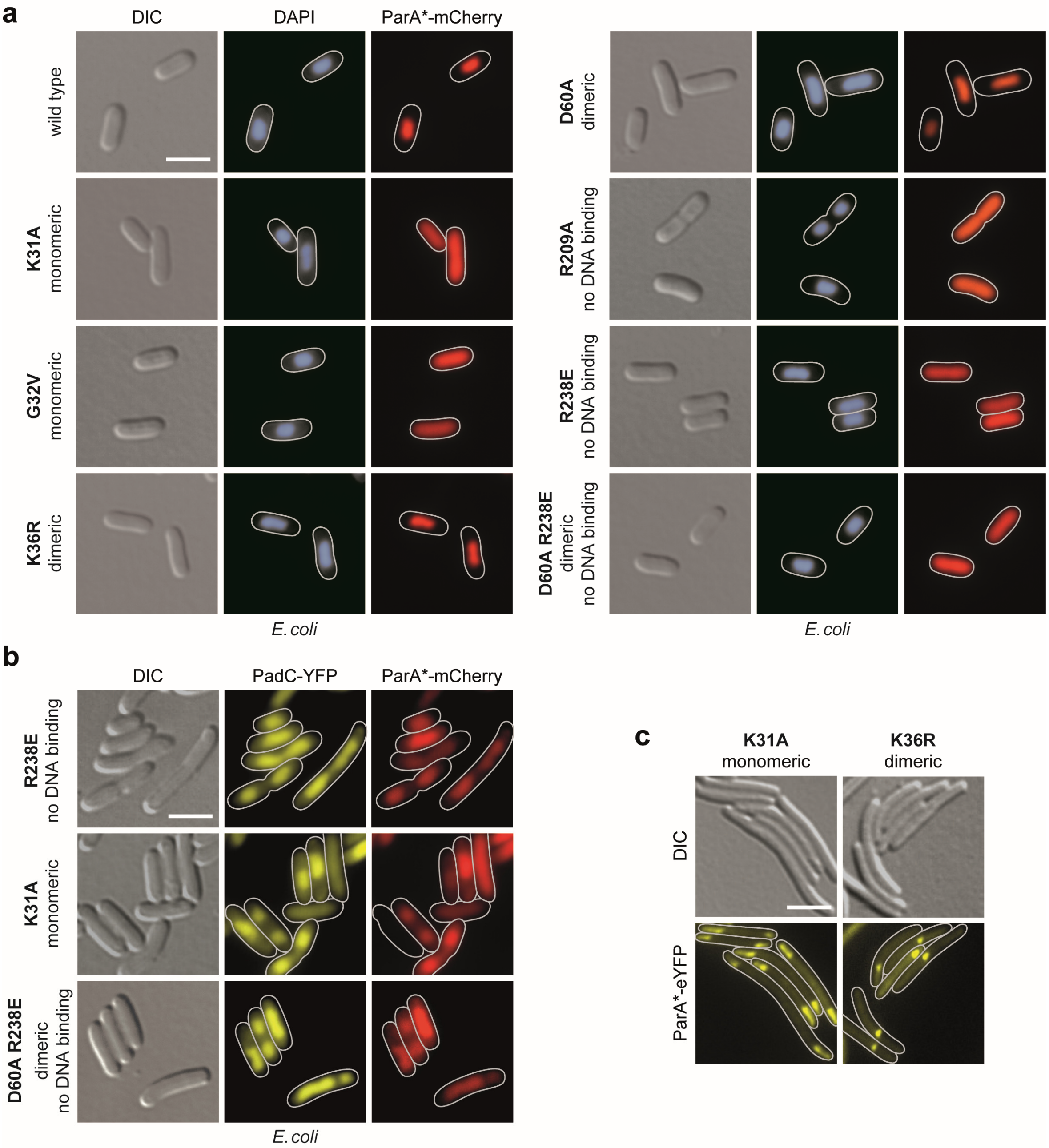

Supplementary Figure 6. PadC interacts with ParA. (a) DNA-binding activity of wild-type and mutant ParA variants upon heterologous expression in *E. coli*. Cells of *E. coli* BL21(DE3) bearing plasmid pLL100 (P_{tet}-*parA-mCherry*) or derivatives thereof encoding the indicated mutant ParA variants (pLL122, pLL124, pLL171, pLL172, pLL173, pLL178, or pLL202) were treated with 0.2 µg/ml aTet (for 1 h) to induce the synthesis of the ParA fusion proteins. Subsequently, 30 µg/ml chloramphenicol was added to induce nucleoid condensation. After 30 min of incubation, DNA was stained with DAPI and cells were analyzed by DIC and fluorescence microscopy (bar: 3 µm). The PCCs determined for the DAPI and ParA*-mCherry signals are 0.91 ± 0.18 (WT, n = 55 cells), 0.72 ± 0.17 (K31A, n = 32 cells), 0.72 ± 0.12 (G32V, n = 37 cells), 0.95 ± 0.03 (K36R, n = 51 cells), 0.97 ± 0.02 (D60A, n = 50 cells), 0.71 ± 0.09 (R209A, n = 51 cells), 0.79 ± 0.08 (R238E, n = 54 cells), and 0.67 ± 0.17 (D60A R238E, n = 42 cells). **(b)** Colocalization of different ParA variants with PadC in *E. coli*. Cells of *E. coli* BL21(DE3) bearing plasmid pLL101 (P_{T7}-*padC-eyfp*) were co-transformed with pLL122 (P_{tet}-*parA*$_{R238E}$-*mCherry*), pLL171 (P_{tet}-*parA*$_{K31A}$-*mCherry*), or pLL202 (P_{tet}-*parA*$_{D60A\ R238E}$-*mCherry*) and induced with 0.5 mM IPTG and/or 0.2 µg/ml aTet before imaging (bar: 3 µm). The PCCs for the PadC-YFP and ParA*-mCherry signals are 0.96 ± 0.06 (R238E, n = 51 cells), 0.93 ± 0.07 (K31A, n = 51 cells), and 0.92 ± 0.06 (D60A R238E, n = 49 cells). **(c)** Subcellular localization of mutant ParA variants in *M. xanthus*. Cells of strains LL210 (P_{van}-*parA*$_{K31A}$-*eyfp*) or LL212 (P_{van}-*parA*$_{K36R}$-*eyfp*) were induced for 5.5 h with 3 µM vanillate and analyzed by DIC and fluorescence microscopy (bar: 3 µm).

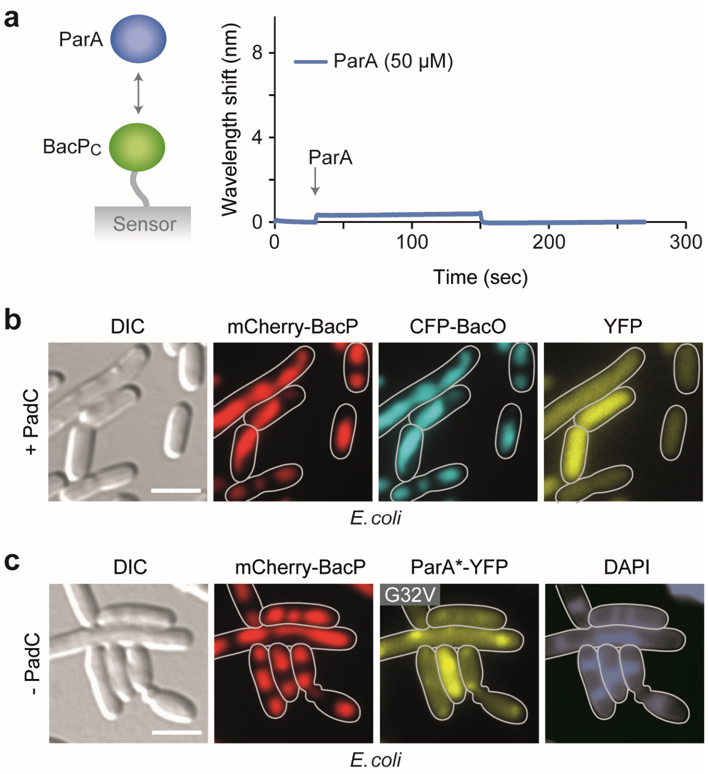

Supplementary Figure 7. PadC is required to recruit ParA to bactofilin complexes. **(a)** Lack of interaction between BacP and ParA. Bio-layer interferometry sensors loaded with $\text{BacP}_{\Delta 1\text{-}115}$ were probed with 50 µM ParA. **(b)** Localization of eYFP in the presence of the BacP·BacO·PadC complex in *E. coli*. Cells of *E. coli* BL21(DE3) were transformed with pLL54 (P_{T7}-*mCherry-bacP cfp-bacO*), pLL205 (P_{T7}-*padC*), and pLL61 (P_{tet}-*eyfp*) and induced with 0.5 mM IPTG (for 3.5 h) and 0.2 µg/ml aTet (for 2.5 h) before imaging (bar: 3 µm). The PCC for the mCherry-BacP and YFP signals is 0.57 ± 0.28 (n = 60 cells). **(c)** Interaction of monomeric ParA_{G32V} with a BacP·BacO complex in the absence of PadC. *E. coli* BL21(DE3) was transformed with pLL54 (P_{T7}-*mCherry-bacP cfp-bacO*) and pLL215 (P_{tet}-*$parA_{G32V}$-eyfp*). Cells were grown in LB medium and induced with 0.5 mM IPTG (for 3.5 h) and 0.2 µg/ml aTet (for 2.5 h) before imaging. The PCC for the mCherry-BacP and ParA_{G32V}-YFP signals is 0.75 ± 0.09 (n = 114 cells).

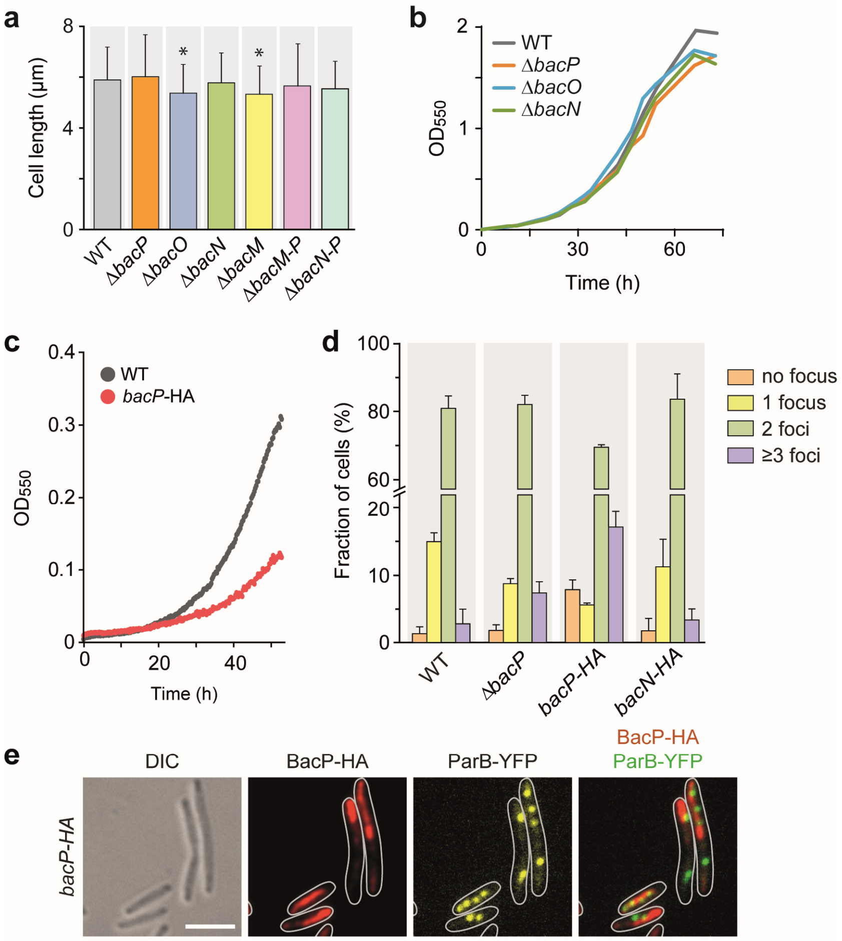

Supplementary Figure 8. A dominant variant of BacP severely affects origin segregation and cell growth. (a) Cell lengths of bactofilin mutants. Cells of strains DK1622 (WT), LL001 (Δ*bacP*), LL002 (Δ*bacO*), LL003 (Δ*bacN*), MT300 (Δ*bacM*), JK328 (Δ*bacMNOP*), and MT295 (Δ*bacNOP*) were visualized by DIC microscopy and analyzed for their lengths. Shown are the average values (± SD; n > 99 cells per strain). Significant differences between the wild-type and the mutant strains are indicated by asterisks ($p < 0.00001$; *t*-test). **(b)** Growth rates of bactofilin-deficient cells. Exponentially growing cultures of strains DK1622 (WT), LL001 (Δ*bacP*), LL002 (Δ*bacO*), and LL003 (Δ*bacN*) were diluted in fresh medium and analyzed for their optical density (OD_{550}) over a period of 75 h. **(c)** Impaired growth of *M. xanthus* in the presence of BacP-HA. Cells of strains DK1622 (WT) and LL032 (*bacP-HA*) were grown to exponential phase and diluted to an OD_{550} of 0.025. Growth was then monitored by following the OD_{550} at intervals of 15 min. **(d)** Increased number of ParB·origin complexes in bactofilin mutants. Strains LL012 (P_{parB}-*parB-eyfp*), LL015 (Δ*bacP* P_{parB}-*parB-eyfp*), LL046 (*bacP-HA* P_{parB}-*parB-eyfp*), and LL040 (*bacN-HA* P_{parB}-*parB-eyfp*) were analyzed by DIC and fluorescence microscopy. Shown are histograms of the number of ParB-YFP foci per cell (± SD; n > 500 cells per strain). **(e)** Abnormal localization of BacP-HA in *M. xanthus*. Cells of strain LL046 (*bacP-HA* P_{parB}-*parB-eyfp*) were analyzed by immunofluoresence microscopy using an anti-HA primary antibodies and an Alexa-Fluor 594-conjugated secondary antibody. In parallel, ParB-YFP was detected by fluorescence microscopy (bar: 3 µm).

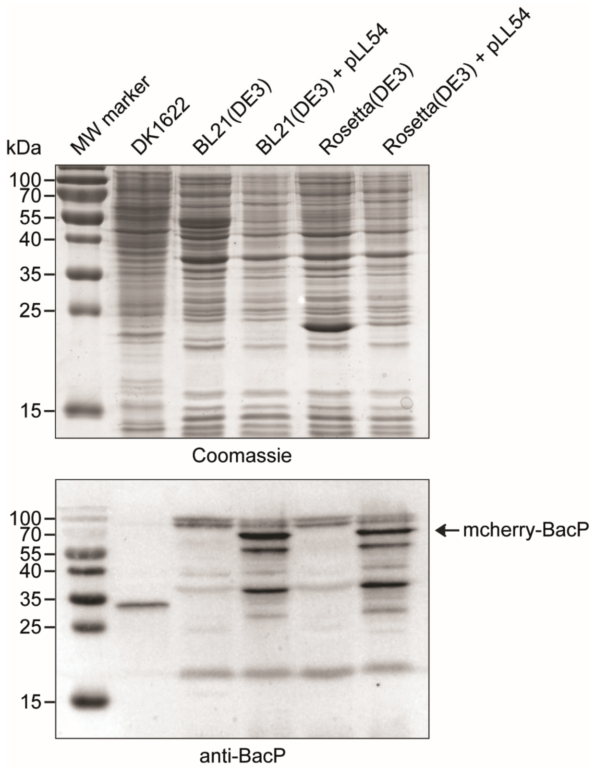

Supplementary Figure 9. Expression levels of fluorescently tagged bactofilin derivatives in *E. coli*. *E. coli* strains BL21(DE3) and Rosetta(DE3)pLysS transformed with plasmid pLL54 (P_{T7}-*mCherry-bacP ecfp-bacO*) were grown to exponential phase and induced for 3.5 h with 0.5 mM IPTG. Lysates of the cells were applied to SDS-polyacrylamide gels and analyzed by Coomassie Blue staining (upper panel) and by immunoblotting with anti-BacP antibodies (lower panel). For comparison, plasmid-free host cells and *M. xanthus* wild-type strain DK1622 were investigated in parallel. Samples were normalized to cell density. A molecular mass standard (in kDa) is shown on the left. The position of the mCherry-BacP fusion is indicated by an arrow.

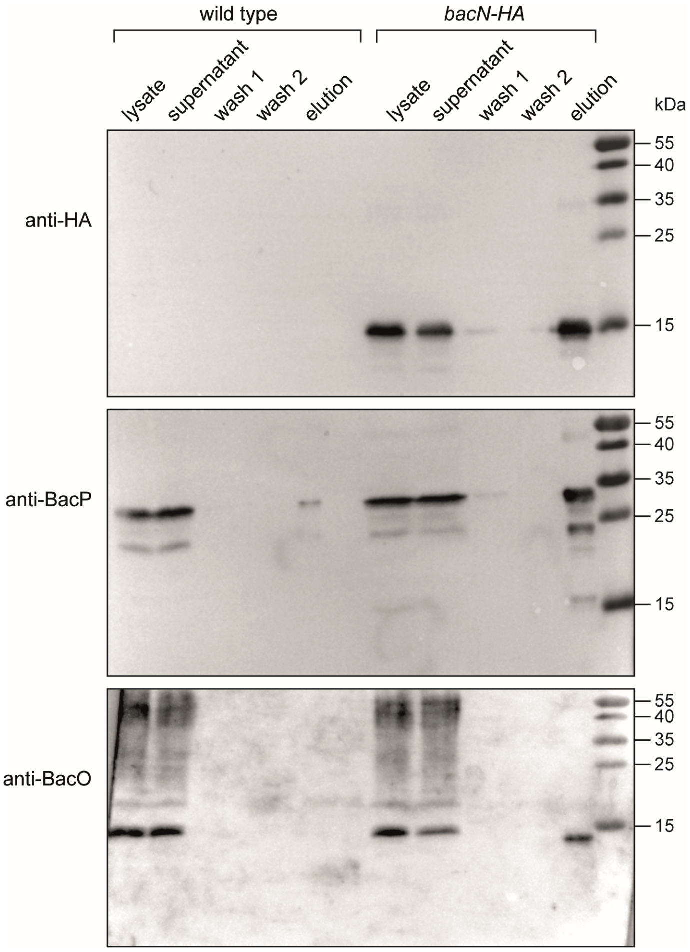

Supplementary Figure 10. Full scans of the Western blots shown in Figure 1e. Cell lysates of strains DK1622 (wild type) and LL033 (BacN-HA) were incubated with anti-HA affinity beads. After isolation of the beads and two washes, interacting proteins were eluted and detected by immunoblot analysis with anti-HA, anti-BacP, and anti-BacO antibodies. Samples of the cell lysates and the supernatants obtained during the isolation and washing steps were analyzed as controls. A molecular mass standard (in kDa) is given on the right.

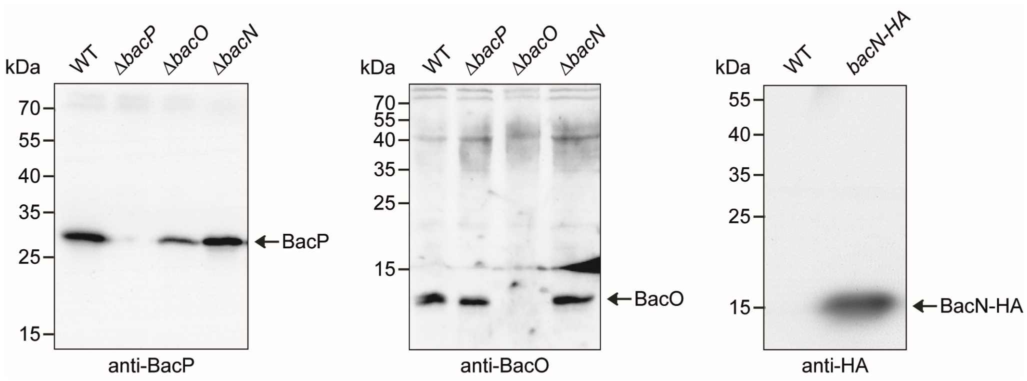

Supplementary Figure 11. Full scans of the Western blots shown in Supplementary Figure 1d. Strains DK1622 (WT), LL001 (Δ*bacP*), LL002 (Δ*bacO*), LL003 (Δ*bacN*), and LL033 (*bacN-HA*) were subjected to immunoblot analysis with anti-BacP, anti-BacO, or anti-HA antibodies. A molecular mass standard (in kDa) is given on the left. The positions of the target proteins are indicated by arrows.

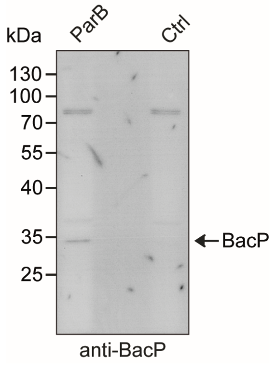

Supplementary Figure 12. Full scan of the Western blot shown in Supplementary Figure 2f. A whole-cell lysate of wild-type strain DK1622 was incubated with beads coupled to purified StrepII-ParB. After isolation of the beads, bound protein was eluted, concentrated with trichloroacetic acid, and subjected to immunoblot analysis with anti-BacP antibodies. A reaction performed with beads not pre-incubated with StrepII-ParB served as a control. A molecular mass standard (in kDa) is given on the left. The position of the BacP signal is indicated by an arrow.

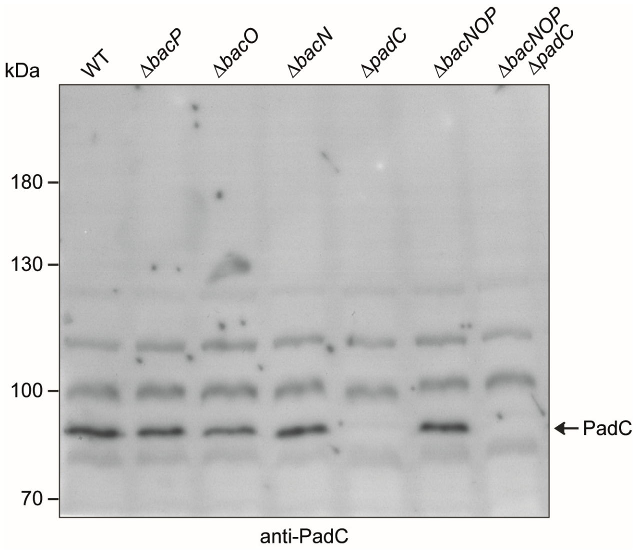

Supplementary Figure 13. Full scan of the Western blot shown in Supplementary Figure 4c. Cells of strains DK1622 (WT), LL001 *(ΔbacP)*, LL002 *(ΔbacO)*, LL003 *(ΔbacN)*, LL101 *(ΔpadC)*, MT295 *(ΔbacNOP)*, and LL174 *(ΔbacNOP ΔpadC)* were subjected immunoblot analysis with anti-PadC antibodies. A molecular mass standard (in kDa) is given on the left. The position of the PadC signal is indicated by an arrow.

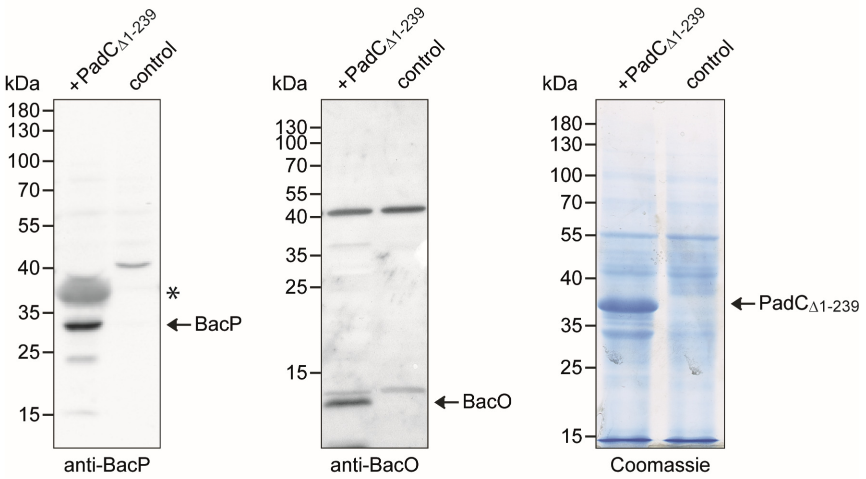

Supplementary Figure 14. Full scans of the Western blots and the Coomassie blue-stained SDS-gel shown in Figure 3g. A whole-cell lysate of wild-type strain DK1622 was incubated with Ni-NTA beads loaded with purified His_6-$PadC_{\Delta 1\text{-}239}$ (+ PadC). After isolation of the beads, bound protein was eluted and subjected to SDS-PAGE and to immunoblot analysis with anti-BacP and anti-BacO antibodies, respectively. A reaction with beads not pre-incubated with purified protein served as a control. A molecular mass standard (in kDa) is given on the left. The positions of the target proteins are indicated by arrows. The asterisk marks a non-specific cross-reaction of the anti-BacP antibody with $PadC_{\Delta 1\text{-}239}$.

SUPPLEMENTARY TABLES

Supplementary Table 1. *M. xanthus* strains used in this study.

Strain	Genotype/description	Construction	Reference
DK1622	*M. xanthus* wild-type strain		Kaiser[3]
MT295	DK1622 *ΔbacNOP*		Kühn *et al.*[4]
MT300	DK1622 *ΔbacM*		Kühn *et al.*[4]
JK328	DK1622 *ΔbacMNOP*		Kühn *et al.*[4]
LL001	DK1622 *ΔbacP*		Bulyha *et al.*[5]
LL002	DK1622 *ΔbacO*		Bulyha *et al.*[5]
LL003	DK1622 *ΔbacN*		Bulyha *et al.*[5]
LL012	DK1622 *attB*::P_{parB}-*parB-eyfp*	Integration of pAH7 in DK1622	This work
LL013	DK1622 *ΔbacM attB*::P_{parB}-*parB-eyfp*	Integration of pAH7 in MT300	This work
LL014	DK1622 *ΔbacN attB*::P_{parB}-*parB-eyfp*	Integration of pAH7 in LL003	This work
LL015	DK1622 *ΔbacP attB*::P_{parB}-*parB-eyfp*	Integration of pAH7 in LL001	This work
LL016	DK1622 *ΔbacMNOP attB*::P_{parB}-*parB-eyfp*	Integration of pAH7 in JK328	This work
LL018	DK1622 *ΔbacO attB*::P_{parB}-*parB-eyfp*	Integration of pAH7 in LL002	This work
LL019	DK1622 *ΔbacNOP attB*::P_{parB}-*parB-eyfp*	Integration of pAH7 in MT295	This work
LL032	DK1622 *bacP-HA*	Gene replacement in DK1622 using pLL9	This work
LL033	DK1622 *bacN-HA*	Gene replacement in DK1622 using pLL11	This work
LL040	DK1622 *bacN-HA attB*::P_{parB}-*parB-eyfp*	Integration of pAH7 in LL033	This work
LL046	DK1622 *bacP-HA attB*::P_{parB}-*parB-eyfp*	Integration of pAH7 in LL032	This work
LL101	DK1622 *ΔpadC*	In-frame deletion of *padC* in DK1622 using pLL38	This work
LL102	DK1622 *ΔpadC attB*::P_{parB}-*parB-eyfp*	Integration of pAH7 in LL101	This work
LL110	DK1622 *ΔpadC bacN-HA*	In-frame deletion of *padC* in LL033 using pLL38	This work
LL116	DK1622 *padC-mCherry*	Gene replacement in DK1622 using pLL72	This work
LL117	DK1622 *ΔbacNOP padC-mCherry*	Gene replacement in MT295 using pLL72	This work
LL118	DK1622 *padC-mCherry attB*::P_{parB}-*parB-eyfp*	Integration of pAH7 in LL116	This work
LL130	DK1622 *ΔbacP padC-mCherry*	Gene replacement in LL001 using pLL72	This work
LL134	DK1622 *attB*::P_{cuoA}-*padC-mCherry*	Integration of pLL74 in DK1622	This work
LL135	DK1622 *ΔbacP padC-mCherry attB*::P_{pilA}-*bacP*	Integration of pLL33 in LL130	This work
LL141	DK1622 *ΔbacO padC-mCherry*	Gene replacement in LL002 using pLL72	This work
LL142	DK1622 *ΔbacN padC-mCherry*	Gene replacement in LL003 using pLL72	This work
LL145	DK1622 *attB*::P_{parA}-*parA-mCherry*	Integration of pAH59 in DK1622	This work
LL147	DK1622 *ΔbacNOP attB*::P_{parA}-*parA-mCherry*	Integration of pAH59 in MT295	This work
LL148	DK1622 *ΔbacO attB*::P_{parA}-*parA-mCherry*	Integration of pAH59 in LL002	This work
LL149	DK1622 *ΔbacN attB*::P_{parA}-*parA-mCherry*	Integration of pAH59 in LL003	This work
LL150	DK1622 *bacP-HA attB*::P_{parA}-*parA-mCherry*	Integration of pAH59 in LL032	This work
LL151	DK1622 *ΔbacM attB*::P_{parA}-*parA-mCherry*	Integration of pAH59 in MT300	This work
LL152	DK1622 *ΔbacP attB*::P_{parA}-*parA-mCherry*	Integration of pAH59 in LL001	This work
LL154	DK1622 *ΔpadC attB*::P_{parA}-*parA-mCherry*	Integration of pAH59 in LL101	This work
LL162	DK1622 *attB*::P_{parA}-*parA-mCherry* P_{cuoA}::P_{cuoA}-*parB-eyfp*	Integration of pAH73 in LL145	This work
LL174	DK1622 *ΔbacNOP ΔpadC*	In-frame deletion of *bacP-padC* in DK1622 using pLL104	This work
LL176	DK1622 *ΔbacNOP ΔpadC attB*::P_{parB}-*parB-eyfp*	Integration of pAH7 in LL174	This work
LL186	DK1622 *ΔbacP MXAN18/19*::P_{van}-*parA-eyfp*	Integration of pLL116 in LL001	This work
LL188	DK1622 *ΔbacP MXAN18/19*::P_{van}-*parA-eyfp attB*::P_{pilA}-*bacP*	Integration of pLL33 in LL186	This work
LL192	DK1622 *ΔpadC attB*::P_{parA}-*parA-mCherry MXAN18/19*::P_{van}-*padC*	Integration of pLL126 in LL154	This work
LL193	DK1622 *MXAN18/19*::P_{van}-*parA*$_{R209A}$-*eyfp*	Integration of pLL130 in DK1622	This work
LL201	DK1622 *attB*::P_{parA}-*parA-mCherry MXAN18/19*::P_{van}-*padC-eyfp*	Integration of pLL143 in LL145	This work
LL210	DK1622 *MXAN18/19*::P_{van}-*parA*$_{K31A}$-*eyfp*	Integration of pLL155 in DK1622	This work
LL211	DK1622 *MXAN18/19*::P_{van}-*parA*$_{G32V}$-*eyfp*	Integration of pLL156 in DK1622	This work
LL212	DK1622 *MXAN18/19*::P_{van}-*parA*$_{K36R}$-*eyfp*	Integration of pLL165 in DK1622	This work
LL218	DK1622 *MXAN18/19*::P_{van}-*parA*$_{D60A}$-*eyfp*	Integration of pLL166 in DK1622	This work

Supplementary Table 2. *E. coli* strains used in this study.

Strain	Genotype	Source
TOP10	F⁻ *mcr*A Δ(*mrr-hsd*RMS-*mcr*BC) Φ80*lac*ZΔM15 Δ*lac*X74 *rec*A1 *ara*D139 Δ(*ara leu*) 7697 *gal*U *gal*K *rps*L (Str^R) *end*A1 *nup*G	Invitrogen
Rosetta(DE3)pLysS	F⁻ *ompT hsdS*$_B$(r_B^- m_B^-) *gal dcm* (DE3) pLysSRARE (Cam^R)	Merck Millipore
BL21(DE3)	F⁻ *omp*T *hsd*SB(rB⁻, mB⁻) *gal dcm* (DE3)	Novagen

Supplementary Table 3. General plasmids used in this work.

Plasmid	Description	Reference/Source
pAH7	pSWU30 carrying P_{parB}-*parB-eyfp*	Treuner-Lange *et al.*[6]
pAH17	pET45b(+) carrying *parA*	Harms *et al.*[7]
pAH59	pSWU30 carrying P_{parA}-*parA-mCherry*	Harms *et al.*[7]
pAH73	pMAT15 carrying *parB-eyfp*	Harms *et al.*[7]
pBJ114	*galK*-containing suicide vector for double homologous recombination in *M. xanthus*, Kan^R	Julien *et al.*[8]
pET28a(+)	Vector for overexpression of N-terminally His_6-tagged proteins , Kan^R	Novagen
pET51b(+)	Vector for overexpression of N-terminally StrepII-tagged proteins, Amp^R	Novagen
pETDuet-1	Vector for coexpression of two target genes, Amp^R	Novagen
pIB145	pET45b(+) carrying *bacP*$_{\Delta aa1\text{-}115}$	Bulyha *et al.*[5]
pMAT4	Vector for genes ligated to *cuoA* promoter integrating at *M. xanthus* chromosomal Mx8 *attB* site, Kan^R	Gomez-Santos *et al.*[9]
pMAT15	Vector for genes ligated to *cuoA* promoter integrating at *M. xanthus PcuoA* site, Kan^R	Harms *et al.*[7]
pMCS-2	Cloning vector	Thanbichler *et al.*[10]
pMR3690	Vector for the expression of a gene under the control of the *vanA* promoter, integrating at the *M. xanthus* Mxan18_19 chromosomal locus, Kan^R	Iniesta *et al.*[11]
pMR3691	Vector for the expression of a gene under the control of the *vanA* promoter, integrating at the *M. xanthus* Mxan18_19 chromosomal locus, tet^R	Iniesta *et al.*[11]
pMT325	pASK-IBA3plus derivative with pBBR1MCS-5 backbone, Amp^R, Gm^R	Thanbichler & Shapiro[12]
pRSFDuet-1	Vector for co-expression of two target genes under the control of P_{T7}, Kan^R	Novagen
pSW17	pET21a(+) carrying *bacO*	Kühn *et al.*[4]
pSW105	Vector for genes ligated to *pilA* promoter integrating at *M. xanthus* chromosomal Mx8 *attB* site, Kan^R	Jakovljevic *et al.*[13]
pSWU30	Vector for integrating at *M. xanthus* chromosomal Mx8 *attB* site, Tet^R	Wu *et al.*[14]
pVCFPN-4	Integration plasmid to fuse 5' end us of a target gene to *cfp* under the control of P_{van}, $Gent^R$	Thanbichler *et al.*[10]
pXCHYC-1	Integration plasmid to fuse 3' end us of a target gene to *mCherry* under the control of P_{xyl}, Strep/$Spec^R$	Thanbichler *et al.*[10]
pXCHYN-2	Integration plasmid to fuse 5' end of a target gene to *mCherry* under the control of P_{xyl}, Kan^R	Thanbichler *et al.*[10]
pXVENN-2	Integration plasmid to fuse 5' end us of a target gene to *venus* under the control of P_{xyl}, Kan^R	Thanbichler *et al.*[10]
pXYFPC-2	Integration plasmid to fuse 3' end us of a target gene to *eyfp* under the control of P_{xyl}, Kan^R	Thanbichler *et al.*[10]
pXYFPN-1	Integration plasmid to fuse 5' end us of a target gene to *eyfp* under the control of P_{xyl}, Strep/$Spec^R$	Thanbichler *et al.*[10]

Supplementary Table 4. Plasmids generated in this work.

Plasmid	Description	Construction
pLL9	pBJ114 derivative for replacing native *bacP* with *bacP-HA*	a) PCR amplification of *bacP-HA* with primers MXAN4635-HA-1 and MXAN4635-HA-2<br>b) digestion of the product with HindIII and BamHI<br>c) PCR amplification of the *bacP* downstream region with primers MXAN4635HA-down-for and MXAN4635HA-down-rev<br>d) digestion of the product with with BamHI and EcoRI<br>e) ligation of the fragments into pBJ114 cut with HindIII and EcoRI
pLL11	pBJ114 derivative for replacing native *bacN* with *bacN-HA*	a) PCR amplification of a fragment including 275 bp of the *bacN* upstream region and *bacN-HA* with primers MXAN4637-HA-1 and MXAN4637-HA-2,<br>b) digestion of the product with HindIII and BamHI<br>c) PCR amplification of the *bacN* downstream region with primers MXAN4637HA-down-for and MXAN4637HA-down-rev<br>d) digestion of the product with BamHI and EcoRI<br>e) ligation of the two fragments into pBJ114 cut with HindIII and EcoRI
pLL17	pXCHYN-2 bearing *bacP*	a) PCR amplification of *bacP* with primers MXAN4635-For and MXAN4635-Rev<br>b) digestion of the product with BglII and NheI<br>c) ligation of the fragment into pXCHYN-2 cut with BglII and NheI
pLL33	pSW105 bearing *bacP*	a) PCR amplification of *bacP* with primers Comp MXAN4635-for and Comp MXAN4635-rev<br>b) digestion of the product with XbaI and HindIII<br>c) ligation of the fragment into pSW105 cut with XbaI and HindIII
pLL38	pBJ114 derivative for generating an in-frame deletion of *padC*	a) PCR amplification of the *padC* downstream region and the last 36 bp of *padC* with primers MXAN4634-down-for and MXAN4634-down-rev<br>b) digestion of the product with BamHI and EcoRI<br>c) PCR amplification of the *padC* upstream region and the first 36 bp of *padC* with primers MXAN4634-up-for and MXAN4634-up-rev<br>d) digestion of the product with BamHI and HindIII<br>e) ligation of the two fragments into pBJ114 cut with HindIII and EcoRI
pLL47	pVCFPN-4 bearing *bacO*	a) PCR amplification of *bacO* with primers MXAN4636-For and MXAN4636-Rev<br>b) digestion of the product with BglII and NheI<br>c) ligation of the fragment into pVCFPN-4 cut with BglII and NheI
pLL48	pETDuet-1 bearing *mCherry-bacP*	a) PCR amplification of *mCherry-bacP* from pLL17 with primers mCherry-For-NdeI and mCherry-MXAN4635-Rev-XhoI<br>b) restriction of the product with NdeI and XhoI<br>c) ligation of the fragment into equally treated pETDuet-1.
pLL51	pETDuet-1 bearing *cfp-bacO*	a) PCR amplification of *cfp-bacO* from pLL47 using primers NcoI-CFP-For and CFP-MXAN4636-Rev-EcoRI<br>b) digestion of the product with NcoI and EcoRI<br>c) ligation of the fragment into pETDuet-1 cut with NcoI and EcoRI
pLL54	pETDuet-1 bearing *mCherry-bacP cfp-bacO*	a) PCR amplification of *mCherry-bacP* from pLL17 using primers mCherry-For-NdeI and mCherry-MXAN4635-Rev-MfeI<br>b) digestion of the product with NdeI and MfeI<br>c) ligation of the fragment into pLL51 cut with NdeI and MfeI
pLL61	pMT325 bearing *eyfp*	a) PCR amplification of *eyfp* from pAH7 with primers XbaI-RBS-eYFP-for and parB-yfp-Rev-XmaI<br>b) digestion of the product with XbaI and XmaI<br>c) ligation of the fragment into pMT325 cut with XbaI and XmaI
pLL64	pXCHYC-1 bearing *padC*	a) amplification of *padC* with primers MXAN4634-new-For-NdeI and MXAN4634-new-Rev-EcoRI<br>b) digestion of the product with NdeI and EcoRI<br>c) ligation of the fragment into pXCHYC-1 cut with NdeI and EcoRI
pLL66	pLL64 carrying the *padC* downstream region	a) amplification of the *padC* downstream region with primers MXAN4634down-for-2 and MXAN4634-down-rev<br>b) digestion of the product with NheI and EcoRI<br>c) ligation of the fragment into pLL64 cut with NheI and EcoRI
pLL72	pBJ114 derivative for replacing native *padC* with *padC-mCherry*	a) PCR amplification of a fragment containing *padC-mCherry* and the *padC* downstream region from pLL66 using primers MXAN4634-For-XbaI and MXAN4634down-Rev-NheI<br>b) digestion of the product with XbaI and NheI, followed by blunting of the fragment with T4 DNA polymerase<br>c) ligation of the product into pBJ114 cut with XbaI and blunted with T4 DNA polymerase
pLL74	pMAT4 bearing *padC-mCherry*	a) PCR amplification of the *padC-mCherry* fragment from pLL72 using primers MXAN4634-For-XbaI and mCherry-Rev-NheI<br>b) digestion of the product with NheI, treatment with T4 DNA polymerase, and subsequent digestion with XbaI<br>c) ligation of the fragment into pMAT4 that had been cut with HindIII, treatment with T4 DNA polymerase, and subsequently digestion with XbaI

Supplementary Table 4. Plasmids generated in this work (continued).

Plasmid	Description	Construction
pLL80	pET51b(+) bearing *parB*	a) PCR amplification of *parB* using primers ParB-for-BamHI strep and ParB-rev-NotI strep<br>b) digestion of the product with BamHI and NotI<br>c) ligation of the fragment into pET51b(+) cut with BamHI and NotI
pLL85	pXYFPC-2 bearing *parA*	a) PCR amplification of *parA* with primers ParA-for-NdeI and ParA-rev-EcoRI<br>b) digestion of the product with NdeI and EcoRI<br>c) ligation of the fragment into pXYFPC-2 cut with NdeI and EcoRI
pLL86	pMT325 bearing *parA-eyfp*	a) PCR amplification of *parA-eyfp* from pLL85 using primers ParA-eyfp-for-XbaI and ParA-eyfp-rev-XmaI<br>b) digestion of the product with XbaI and XmaI<br>c) ligation of the fragment into pMT325 cut with XbaI and XmaI
pLL87	pXYFPC-2 bearing *padC*	a) PCR amplification of *padC* with primers MXAN4634-For-new-NdeI and MXAN4634-Rev-new-EcoRI<br>b) digestion of the product with NdeI and EcoRI<br>c) ligation of the fragment into pXYFPC-2 cut with NdeI and EcoRI
pLL89	pSWU30 bearing P_{parA}-*parA-eyfp*	a) amplification of *eyfp* from pXYFPC-2 using primers eYFP-for-BamHI and eYFP-rev-HindIII<br>b) digestion of the product with BamHI and HindIII<br>c) ligation of the fragment into pAH59 cut with BamHI and HindIII
pLL100	pMT325 bearing *parA-mCherry*	a) PCR amplification of *parA-mCherry* from pAH59 using primers ParA-eyfp-for-XbaI and mCherry-rev-HindIII<br>b) digestion of the product with XbaI and HindIII<br>c) ligation of the fragment into pMT325 cut with XbaI and HindIII
pLL101	pRSFDuet-1 bearing *padC-eyfp*	a) PCR amplification of *padC-eyfp* from pLL87 using primers MXAN4634-For-new-NdeI and eYFP-rev-NheI<br>b) digestion of the product with NheI and treatment with T4 DNA polymerase<br>c) digestion of pRSFDuet-1 with KpnI, followed by the treatment with T4 DNA polymerase<br>d) digestion of both the *padC-eyfp* fragment and linearized pRSFDuet-1 with NdeI<br>e) ligation of the two restriction products
pLL104	pBJ114 derivative for generating an in-frame deletion of MXAN4634-7	a) PCR amplification of a fragment containing 700 bp of the *padC* downstream region and the last 36 bp of *padC* with primers MXAN4634down-1-HindIII and MXAN4634down-2-BamHI<br>b) digestion of the product with HindIII and BamHI<br>c) PCR amplification of a fragment containing 736 bp of the *bacN* upstream region and the first 36 bp of *bacN* with primers BacN-up-1-BamHI and BacN-up-2-EcoRI<br>d) digestion of the product with BamHI and EcoRI<br>e) ligation of the two fragments into HindIII/EcoRI-treated pBJ114
pLL105	pET28a(+) bearing $padC_{\Delta AA1\text{-}239}$	a) PCR amplification of $padC_{\Delta AA1\text{-}239}$ with primers mxan4634c-for and mxan4634c-rev<br>b) digestion of the product with NdeI and EcoRI<br>c) ligation of the fragment into NdeI/EcoRI-treated pET28a(+)
pLL116	pMR3691 bearing *parA-eyfp*	a) PCR amplification of *parA-eyfp* from pLL85 with primers ParA-for-NdeI and eYFP-rev-NheI<br>b) digestion of the product with NdeI and NheI<br>c) ligation of the fragment into NdeI/NheI-treated pMR3691
pLL118	pXVENN-2 bearing $padC_{\Delta AA1\text{-}239}$	a) PCR amplification of $padC_{\Delta AA1\text{-}239}$ with primers MXAN4634c-for-BglII and mxan4634c-rev<br>b) digestion of the product with BglII and EcoRI<br>c) ligation of the fragment into pXVENN-2 cut with BglII and EcoRI
pLL119	pXYFPC-2 bearing $parA_{R209A}$	site-directed mutagenesis of pLL85 with primers parA(R209A)-for and parA(R209A)-rev
pLL120	pRSFDuet-1 bearing *venus*-$padC_{\Delta AA1\text{-}239}$	a) PCR amplification of *venus*-$padC_{\Delta AA1\text{-}239}$ from pLL118 using primers NcoI-CFP-For and mxan4634c-rev<br>b) restriction of the product with NcoI and EcoRI<br>c) ligation of the fragment into pRSFDuet-1 cut with NcoI and EcoRI
pLL121	pXCHYC-1 bearing $parA_{R238E}$	a) site-directed mutagenesis of pLL85 with primers parA(R238E)-for and parA(R238E)-rev<br>b) release of the $parA_{R238E}$ gene by digestion with NdeI and EcoRI<br>c) ligation of $parA_{R238E}$ into NdeI/EcoRI-treated pXCHYC-1
pLL122	pMT325 bearing $parA_{R238E}$-*mCherry*	a) PCR amplification of $parA_{R238E}$-*mCherry* from pLL121 using primers ParA-eyfp-for-XbaI and mCherry-rev-HindIII<br>b) digestion of the product with XbaI and HindIII<br>c) ligation of the fragment into XbaI/HindIII-treated pMT325
pLL123	pXCHYC-1 bearing $parA_{R209A}$	a) PCR amplification of $parA_{R209A}$ from pLL119A with primers ParA-for-NdeI and ParA-rev-EcoRI<br>b) ligation of $parA_{R209A}$ into NdeI/EcoRI-treated pXCHYC-1
pLL124	pMT325 bearing $parA_{R209A}$-*mCherry*	a) PCR amplification of $parA_{R209A}$-mCherry from pLL123 using primers ParA-eyfp-for-XbaI and mCherry-rev-HindIII<br>b) digestion of the product with XbaI and HindIII<br>c) ligation of the fragment into pMT325 cut with XbaI and HindIII

Supplementary Table 4. Plasmids generated in this work (continued).

Plasmid	Description	Construction
pLL126	pMR3690 bearing *padC*	a) PCR amplification of *padC* with primers MXAN4634-For-new-NdeI and mxan4634c-rev<br>b) digestion of the product with NdeI and EcoRI<br>c) ligation of the fragment into pMR3690 cut with NdeI and EcoRI
pLL130	pMR3691 bearing $parA_{R209A}$-*eyfp*	a) PCR amplification of $parA_{R209A}$-*eyfp* from pLL119 with primers ParA-for-NdeI and eYFP-rev-NheI<br>b) digestion of the product with NdeI and NheI<br>c) ligation of the fragment into NdeI/NheI-treated pMR3691
pLL137	pRSFDuet-1 bearing *eyfp*	a) PCR amplification of *eyfp* from pLL116 with primers NcoI-CFP-For and eYFP-rev-HindIII,<br>b) digestion of the product with NcoI and HindIII<br>c) ligation of the fragment into pRSFDuet-1 cut with NcoI and HindIII
pLL143	pMR3690 bearing *padC-eyfp*	a) release of *padC-eyfp* from pLL87 by digestion with NdeI and NheI<br>b) ligation of the product into NdeI/NheI-treated pMR3690
pLL147	pXYFPC-2 bearing $parA_{K31A}$	site-directed mutagenesis of pLL85 with primers parA(K31A)-for and parA(K31A)-rev
pLL148	pXYFPC-2 bearing $parA_{G32V}$	site-directed mutagenesis of pLL85 with primers parA(G32V)-for and parA(G32V)-rev
pLL150	pXYFPC-2 bearing $parA_{K36R}$	site-directed mutagenesis of pLL85 with primers parA(K36R)-for and parA(K36R)-rev
pLL151	pXYFPC-2 bearing $parA_{D60A}$	site-directed mutagenesis of pLL85 with primers parA(D60A)-for and parA(D60A)-rev
pLL155	pMR3691 bearing $parA_{K31A}$-*eyfp*	a) PCR amplification of parAK31A-eyfp from pLL147 with primers ParA-for-NdeI and eYFP-rev-NheI<br>b) digestion of the product with NdeI and NheI<br>c) ligation of the fragment into pMR3691 cut with NdeI and NheI
pLL156	pMR3691 bearing $parA_{G32V}$-*eyfp*	a) PCR amplification of $parA_{G32V}$-*eyfp* from pLL148 with primers ParA-for-NdeI and eYFP-rev-NheI<br>b) digestion of the product with NdeI and NheI<br>c) ligation of the fragment into pMR3691 cut with NdeI and NheI
pLL157	pXCHYC-1 bearing $parA_{K31A}$	a) PCR amplification of $parA_{K31A}$ from pLL147 with primers ParA-for-NdeI and ParA-rev-EcoRI<br>b) digestion of the product with NdeI and EcoRI<br>c) ligation of the fragment into pXCHYC-1 cut with NdeI and EcoRI
pLL158	pXCHYC-1 bearing $parA_{G32V}$	a) PCR amplification of $parA_{G32V}$ from pLL148 with primers ParA-for-NdeI and ParA-rev-EcoRI<br>b) digestion of the product with with NdeI and EcoRI<br>c) ligation of the fragment into pXCHYC-1 cut with NdeI and EcoRI
pLL159	pXCHYC-1 bearing $parA_{K36R}$	a) PCR amplification of $parA_{K36R}$ from pLL150 with primers ParA-for-NdeI and ParA-rev-EcoRI<br>b) digestion of the product with NdeI and EcoRI<br>c) ligation of the fragment into pXCHYC-1 cut with NdeI and EcoRI
pLL163	pXCHYC-1 bearing $parA_{D60A}$	a) PCR amplification of $parA_{D60A}$ from pLL151 with primers ParA-for-NdeI and ParA-rev-EcoRI<br>b) digestion of the product with NdeI and EcoRI<br>c) ligation of the fragment into pXCHYC-1 cut with NdeI and EcoRI
pLL165	pMR3691 bearing $parA_{K36R}$-*eyfp*	a) PCR amplification of $parA_{K36R}$-*eyfp* from pLL150 with primers ParA-for-NdeI and eYFP-rev-NheI<br>b) digestion of the product with NdeI and NheI<br>c) ligation of the fragment into pMR3691 cut with NdeI and NheI
pLL166	pMR3691 bearing $parA_{D60A}$-*eyfp*	a) PCR amplification of $parA_{D60A}$-*eyfp* from pLL151 with primers ParA-for-NdeI and eYFP-rev-NheI<br>b) digestion of the product with NdeI and NheI<br>c) ligation of the fragment into pMR3691 cut with NdeI and NheI
pLL171	pMT325 bearing $parA_{K31A}$-*mCherry*	a) PCR amplification of $parA_{K31A}$-*mCherry* from pLL157 using primers ParA-eyfp-for-XbaI and mCherry-rev-HindIII<br>b) digestion of the product with XbaI and HindIII<br>c) ligation of the fragment into pMT325 cut with XbaI and HindIII
pLL172	pMT325 bearing $parA_{G32V}$-*mCherry*	a) PCR amplification of $parA_{G32V}$-*mCherry* from pLL158 using primers ParA-eyfp-for-XbaI and mCherry-rev-HindIII<br>b) digestion of the product with XbaI and HindIII<br>c) ligation of the fragment into pMT325 cut with XbaI and HindIII
pLL173	pMT325 bearing $parA_{K36R}$-*mCherry*	a) PCR amplification of $parA_{K36R}$-*mCherry* from pLL159 using primers ParA-eyfp-for-XbaI and mCherry-rev-HindIII<br>b) digestion of the product with XbaI and HindIII<br>c) ligation of the fragment into pMT325 cut with XbaI and HindIII
pLL178	pMT325 bearing $parA_{D60A}$-*mCherry*	a) PCR amplification of $parA_{D60A}$-*mCherry* from pLL163 using primers ParA-eyfp-for-XbaI and mCherry-rev-HindIII<br>b) digestion of the product with XbaI and HindIII<br>c) ligation of the fragment into pMT325 cut with XbaI and HindIII
pLL200	pXCHYC-1 bearing $parA_{D60AR238E}$	site-directed mutagenesis of pLL163 with primers parA(R238E)-for and parA(R238E)-rev
pLL202	pMT325 bearing $parA_{D60AR238E}$-*mCherry*	a) PCR amplification of $parA_{D60AR238E}$-*mCherry* from pLL200 using primers ParA-eyfp-for-XbaI and mCherry-rev-HindIII<br>b) digestion of the product with XbaI and HindIII<br>c) ligation of the fragment into pMT325 cut with XbaI and HindIII

Supplementary Table 4. Plasmids generated in this work (continued).

Plasmid	Description	Construction
pLL204	pXYFPC-2 bearing *parA*$_{D60AR238E}$	a) PCR amplification of *parA*$_{D60AR238E}$ from pLL200 with primers ParA-for-NdeI and ParA-rev-EcoRI<br>b) digestion of the product with NdeI and EcoRI<br>c) ligation of the fragment into pXYFPC-2 cut with NdeI and EcoRI
pLL205	pRSFDuet-1 bearing *padC*	a) PCR amplification of *padC* using primers MXAN4634-For-new-NdeI and mxan4634c-rev<br>b) digestion of the product with EcoRI and treatment with T4 DNA polymerase<br>c) digestion of pRSFDuet-1 with KpnI and treatment with T4 DNA polymerase<br>d) digestion of both the *padC* fragment and linearized pRSFDuet-1 with NdeI<br>e) ligation of the two restriction products
pLL215	pMT325 bearing *parA*$_{G32V}$-*eyfp*	a) PCR amplification of *parA*$_{G32V}$-*eyfp* from pLL148 using primers ParA-eyfp-for-XbaI and ParA-eyfp-rev-XmaI<br>b) digestion of the product with XbaI and XmaI<br>c) ligation of the fragment into pMT325 cut with XbaI and XmaI
pLL235	pRSFDuet-1 bearing *ibpA-yfp*	a) PCR amplification of *ibpA* from *E. coli* TOP10 with primers IbpA-1 and IbpA-2<br>b) Fusion of the PCR product with NdeI/EcoRI-treated pXYFPC-2 by Gibson assembly<br>c) PCR amplification of *ibpA-eyfp* from the resulting plasmid using primers IbpA-yfp-g-5 and IbpA-yfp-g-6<br>d) Fusion of the PCR product with NdeI/KpnI-treated pRSFDuet-1 by Gibson assembly
pMO002	pET28a(+) bearing *padC*$_{\Delta AA1-281}$	a) PCR amplification of *padC*$_{\Delta AA1-281}$ with primers MXAN4634c3-for and MXAN4634c-rev<br>b) digestion of the product with NdeI and EcoRI<br>c) ligation of the fragment into NdeI/EcoRI-treated pET28a(+)
pPS17	pXYFPN-2 bearing *bacN*	a) PCR amplification of *bacN* using primers MXAN4637-for-2 and MXAN4637-rev-2<br>b) digestion of the product with EcoRI and BamHI<br>c) ligation of the fragment into pXYFPN-2 cut with EcoRI and BamHI
pPS20	pMT325 bearing *eyfp-bacN*	a) PCR amplification of *eyfp-bacN* from pPS17 using primers YFP-MXAN4637-For and YFP-MXAN4637-Rev<br>b) digestion of the product with BamHI and XmaI<br>c) ligation of the fragment into pMT325 cut with BamHI and XmaI

Supplementary Table 5. Oligonucleotides in this work.

Oligonucleotide	Sequence
BacN-up-1-BamHI	AATTGGATCCGCCCTTGCCGATGATGC
BacN-up-2-EcoRI	AATTGAATTCGGCCACGGTAAGGCCGA
CFP-MXAN4636-Rev-EcoRI	AGCTGAATTCTCAGCGCTCCTTCATGTCCATGTC
Comp MXAN4635-for	TTATCTAGAATGGCCACCGCGAAGGAGCTCTCA
Comp MXAN4635-Rev	TTAAAGCTTCTAGCGGGTCTTCTTCTTCACCAC
eYFP-for-BamHI	AATTGGATCCATGGTGAGCAAGGGCGA
eYFP-rev-HindIII	GGCCAAGCTTTTACTTGTACAGCTCGTC
eyfp-rev-NheI	AGCTGCTAGCTTACTTGTACAGCTCGTC
IbpA-1	TTGGGGAGACGACCATATGATGCGTAACTTTGATTTATCCCCGC
IbpA-2	GACGCGTAACGTTCGAATTCGCGTTGATTTCGATACGGCG
IbpA-yfp-g-5	AGAAGGAGATATACATATGCGTAACTTTGATTTATCCCCGCTTTACC
IbpA-yfp-g-6	CAGACTCGAGGGTACCTTACTTGTACAGCTCGTCCATGCC
mCherry-4635-Rev-XhoI	AATTCTCGAGCTAGCGGGTCTTCTTCTTCACCA
mCherry-For-NdeI	AGCTCATATGATGGTGAGCAAGGGCGAGGAG
mCherry-MXAN4635N-rev	AATTCAATTGCTACAGACGTCCCGGCTCCACGTC
mCherry-MXAN4635rev-MfeI	AATTCAATTGCTAGCGGGTCTTCTTCTTCACCA
mCherry-rev-HindIII	ATAAGCTTTTACTTGTACAGCTCGTCCATGCCGCCG
mCherry-Rev-NheI	TATAGCTAGCTTACTTGTACAGCTCGTCCATGCCGCCG
mxan4634c-for	AATTCATATGCCCTCCGACGGCGAGGCCCG
MXAN4634c-for-BglII	AATTAGATCTCCCTCCGACGGCGAGGCC
mxan4634c-rev	AGTCGAATTCTTACAGCCCCTCCAGATAGG
MXAN4634down-1-HindIII	GAGCAAGCTTAGAATCTTCCGGTAGGA
MXAN4634down-2-BamHI	AATTGGATCCTCGTCCGAGCTGGTCAC
MXAN4634-down-for	TAGGATCCTCGTCCGAGCTGGTCACCTATCTGG
MXAN4634down-For-2	TATAGCTAGCGCCATGGCGGAACCTCTCGTGCG
MXAN4634-down-rev	TTAAGAATTCCTCGCGGAAGGACAGCGTCTGCAGCGTC
MXAN4634down-Rev-NheI	TTAAGCTAGCCTCGCGGAAGGACAGCGTCTG
MXAN4634-For-new-NdeI	TATACATATGCGGGCGCGTGGGCTTTTCAGGG
MXAN4634-For-XbaI	TTAATCTAGAATGCGGGCGCGTGGGCTTTTC
MXAN4634-Rev-new-EcoRI	TAGAATTCGACAGCCCCTCCAGATAGGTGACCAG
MXAN4634-up-For	TATAAAGCTTCCTCCTTCAAGGGCAACGTCGAC
MXAN4634-up-Rev	TAGGATCCCATGGTGGCACCCCTGAAAAGCCCA
MXAN4634c3-for	AATTCATATGCCCGAACCCGAGCAGCGCG
MXAN4635-For	TATAGATCTATGGCCACCGCGAAGGAGCTCTCAG
MXAN4635-HA-1	TATAAAGCTTGTGGCCACCGCGAAGGAGCTCTCAG
MXAN4635-HA-2	TAGGATCCCTAAGCGTAGTCTGGGACGTCGTATGGGTAGCGGGTCTTCTTCTTCACCA
MXAN4635HA-down-for	TAGGATCCCGCGCACCGCCCGCGCCGCCAGTCG
MXAN4635HA-down-rev	TTAAGAATTCACTGCTCGGCGCGCGCCTCCTCGTT
MXAN4635-Rev	TTTTGCTAGCCTAGCGGGTCTTCTTCTTCACCACG
MXAN4636-For	TATAGATCTATGAGCTTCACGCCGCGCACGGCACG
MXAN4636-Rev	TTTTGCTAGCTCAGCGCTCCTTCATGTCCATGTC
MXAN4637-for-2	GCGCGAATTCGGCAACGGGTGAAACGGGCA
MXAN4637-HA-1	TATAAAGCTTCGTGCTGCTGGCGCCGAAGATTCAA
MXAN4637-HA-2	TAGGATCCCTAAGCGTAGTCTGGGACGTCGTATGGGTAAATGTCGTCAGGAAGCCGTAC
MXAN4637HA-down-for	TAGGATCCCGGCTTCCTGACGACATTTGAGCTTCAC
MXAN4637HA-down-rev	TTAAGAATTCGGCAGCGGCTTGCTTCCCAGGCCAC
MXAN4637-rev-2	GCGCGGATCCTCAAATGTCGTCAGGAAGCC
NcoI-CFP-For	AATTCCATGGTGAGCAAGGGCGAGGAGCTGTTCA
parA(D60A)-for	CTGGTGGACATGGCCCCGCAGGGCAAC
parA(D60A)-rev	GTTGCCCTGCGGGGCCATGTCCACCAG
parA(G32V)-for	CTTCCCGACGCCGACCTTCTGGTTGGA
parA(G32V)-rev	TCCAACCAGAAGGTCGGCGTCGGGAAG
parA(K31A)-for	CATCTCCAACCAGGCGGGCGGCGTCGGG
parA(K31A)-REV	CCCGACGCCGCCCGCCTGGTTGGAGATG
parA(K36R)-for	CGGTGGTGGTCCTCCCGACGCCG
parA(K36R)-rev	CGGCGTCGGGAGGACCACCACCG
parA(R209A)-for	ACCATGTTCGACTCGGCGGCGAACATTGCCCA
parA(R209A)-rev	TGGGCAATGTTCGCCGCCGAGTCGAACATGGT
parA(R238E)-for	GTGCCGCGCAACGTGGAGCTGTCCGAGTGCCCC
parA(R238E)-rev	GGGGCACTCGGACAGCTCCACGTTGCGCGGCAC
ParA-eyfp-for-XbaI	AATTCTAGAAGGAGGAATTCACCATGCACTGCATCACGCGCGGGGCC
ParA-eyfp-rev-XmaI	AATTCCCGGGTTACTTGTACAGCTCGTCCAT

Supplementary Table 5. Oligonucleotides in this work (continued).

Oligonucleotide	Sequence
ParA-for-NdeI	AATTCATATGCACTGCATCACGCGCGGG
ParA-rev-EcoRI	AATTGAATTCCCAGCCACGCGCCTGCGA
ParB-for-BamHI strep	TGTAGGATCCGATGGTGAAAGCAGACATG
ParB-rev-NotI strep	AATTGCGGCCGCCTACTCCTTCCTGAGAAGC
parB-yfp-rev-XmaI	GCGCCCCGGGTTACTTGTACAGCTCGTCCATGCCG
XbaI-RBS-eYFP-for	TCTAGAAGGAGGAATTCACCATGGTGAGCAAGGGCGAGGAGC
YFP-MXAN4637-For	GCGCCCCGGGAGGAGGAATTCACCATGGTGAGCAAGGGCGAGGAGCTGT
YFP-MXAN4637-Rev	GCGCGGATCCTCAAATGTCGTCAGGAAGCCG

SUPPLEMENTARY REFERENCES

1. Lindner, A. B., Madden, R. Demarez, A., Stewart, E. J. & Taddei, F. Asymmetric segregation of protein aggregates is associated with cellular aging and rejuvenation. *Proc. Natl. Acad. Sci. U S A* **105**, 3076-3081 (2008).
2. Oberto, J. SyntTax: a web server linking synteny to prokaryotic taxonomy. *BMC Bioinformatics* **16**, 4 (2013).
3. Kaiser, D. Social gliding is correlated with the presence of pili in *Myxococcus xanthus*. *Proc. Natl. Acad. Sci. USA* **76**, 5952-5956 (1979).
4. Kühn, J., Briegel, A., Mörschel, E., Kahnt, J., Leser, K., Wick, S., Jensen, G. J., and Thanbichler, M. Bactofilins, a ubiquitous class of cytoskeletal proteins mediating polar localization of a cell wall synthase in *Caulobacter crescentus*. *EMBO J.* **29**, 327-339 (2010).
5. Bulyha, I., Lindow, S., Lin, L., Bolte, K., Wuichet, K., Kahnt, J., van der Does, C., Thanbichler, M., and Søgaard-Andersen, L. Two small GTPases act in concert with the bactofilin cytoskeleton to regulate dynamic bacterial cell polarity. *Dev. Cell* **25**, 119-131 (2013).
6. Treuner-Lange, A., Aguiluz, K., van der Does, C., Gomez-Santos, N., Harms, A., Schumacher, D., Lenz, P., Hoppert, M., Kahnt, J., Munoz-Dorado, J. *et al.* PomZ, a ParA-like protein, regulates Z-ring formation and cell division in *Myxococcus xanthus*. *Mol. Microbiol.* **87**, 235-253 (2013).
7. Harms, A., Treuner-Lange, A., Schumacher, D., and Søgaard-Andersen, L. Tracking of chromosome and replisome dynamics in *Myxococcus xanthus* reveals a novel chromosome arrangement. *PLoS Genet.* **9**, e1003802 (2013).
8. Julien, B., Kaiser, A. D., and Garza, A. Spatial control of cell differentiation in *Myxococcus xanthus*. *Proc. Natl. Acad. Sci. USA* **97**, 9098-9103 (2000).
9. Gomez-Santos, N., Treuner-Lange, A., Moraleda-Munoz, A., Garcia-Bravo, E., Garcia-Hernandez, R., Martinez-Cayuela, M., Perez, J., Sogaard-Andersen, L., and Munoz-Dorado, J. Comprehensive set of integrative plasmid vectors for copper-inducible gene expression in *Myxococcus xanthus*. *Appl. Environ. Microbiol.* **78**, 2515-2521 (2012).
10. Thanbichler, M., Iniesta, A. A. & Shapiro, L. A comprehensive set of plasmids for vanillate- and xylose-inducible gene expression in *Caulobacter crescentus*. *Nucleic Acids Res.* **35**, e137 (2007).
11. Iniesta, A. A., Garcia-Heras, F., Abellon-Ruiz, J., Gallego-Garcia, A., and Elias-Arnanz, M. Two systems for conditional gene expression in *Myxococcus xanthus* inducible by isopropyl-beta-D-thiogalactopyranoside or vanillate. *J. Bacteriol.* **194**, 5875-5885 (2012).
12. Thanbichler, M. & Shapiro, L. MipZ, a spatial regulator coordinating chromosome segregation with cell division in *Caulobacter*. *Cell* **126**, 147-162 (2006).
13. Jakovljevic, V., Leonardy, S., Hoppert, M., and Søgaard-Andersen, L. PilB and PilT are ATPases acting antagonistically in type IV pilus function in *Myxococcus xanthus*. *J. Bacteriol.* **190**, 2411-2421 (2008).
14. Wu, S. S., Wu, J. & Kaiser, D. The *Myxococcus xanthus pilT* locus is required for social gliding motility although pili are still produced. *Mol. Microbiol.* **23**, 109-121 (1997).
